# N^6^-methyladenosine modification of the *Aedes aegypti* transcriptome and its alteration upon dengue virus infection in Aag2 cell line

**DOI:** 10.1038/s42003-022-03566-8

**Published:** 2022-06-20

**Authors:** Zhenkai Dai, Kayvan Etebari, Sassan Asgari

**Affiliations:** Australian Infectious Disease Research Centre, School of Biological Sciences, The University of Queensland, Brisbane, Queensland Australia

**Keywords:** Entomology, Virology

## Abstract

The N^6^-methyladenosine (m^6^A) modification of RNA has been reported to affect viral infections. Studies have confirmed the role of m^6^A in replication of several vector-borne flaviviruses, including dengue virus (DENV), in mammalian cells. Here, we explored the role of m^6^A in DENV replication in the mosquito *Aedes aegypti* Aag2 cell line. We first determined the presence of m^6^A on the RNAs from mosquito cells and using methylated RNA immunoprecipitation and sequencing (MeRIP-Seq) identified m^6^A modification of the mosquito transcriptome and those that changed upon DENV infection. Depletion of m^6^A methyltransferases and the m^6^A binding protein YTHDF3 RNAs decreased the replication of DENV. In particular, we found that the *Ae. aegypti ubiquitin carrier protein 9* (*Ubc9*) is m^6^A modified and its expression increases after DENV infection. Silencing of the gene and ectopic expression of Ubc9 led to reduced and increased DENV replication, respectively. The abundance of *Ubc9* mRNA and its stability were reduced with the inhibition of m^6^A modification, implying that m^6^A modification of *Ubc9* might enhance expression of the gene. We also show that the genome of DENV is m^6^A modified at five sites in mosquito cells. Altogether, this work reveals the involvement of m^6^A modification in *Ae. aegypti*-DENV interaction.

## Abstract (web summary)

Analysis of m^6^A RNA modifications in the mosquito transcriptome and their changes upon dengue virus infection provides insight into the role of epigenetics in regulating viral replication in mosquitoes.

## Introduction

Nucleotides in RNA molecules are known to be modified by more than 100 different modifications, however, N^6^-methyladenosine (m^6^A) is the most common RNA modification, particularly in mRNAs and long non-coding RNAs^1,2^. m^6^A affects almost all aspects of mRNA metabolism, including splicing, translation, stability and maturation^3–5^. Recent findings have indicated that m^6^A methylation is not static, but a dynamic and reversible modification in RNA^2,6^. There are three sets of proteins involved in dynamic m^6^A modification: writers, readers, and erasers. m^6^A writers are responsible for RNA methylation and form a multiprotein complex consisting of methyltransferase-like 3 (METTL3), METTL14, and Wilms’ tumour 1-associated protein (WTAP), in which METTL3 has a catalytic function, METTL14 has an RNA-binding property, and WTAP is a stabilizing factor^7^. The METTL3-METTL14-WTAP complex targets the consensus motif DRACH (where D = G/A/U, R = G/A and H = U/A/C) in mRNAs^8–10^. m^6^A modification can be removed by m^6^A erasers or demethylases such as fat mass and obesity-associated protein (FTO) and ALKB homologue 5 (ALKBH5) protein^11,12^. Finally, m^6^A readers, that specifically bind to m^6^A, mediate m^6^A modification functions that range from blocking or inducing protein-RNA interactions or facilitating subsequent reactions such as alternative splicing, promoting translation of m^6^A-modified mRNA or targeting mRNA for degradation^2^. The major m^6^A readers in the nucleus are heterogeneous nuclear ribonucleoprotein C (HNRNPC) and HNRNPA2B1, and in the cytoplasm, YTH-domain family 1 (YTHDF1) and YTHDF2 proteins^13,14^.

m^6^A analysis is a relatively new area of research and the relevant literature on insects is currently scant and almost non-existent on mosquitoes; except one, in which m^6^A modification of mRNAs in *Aedes albopictus* was shown in 1977 using a biochemical approach^15^. More recently, m^6^A RNA modification was shown in *Drosophila melanogaster* and its role in neural function and sex determination was established^3^. Further, m^6^A has also been shown in the silkworm, *Bombyx mori*. Knocking down *BmMETTL3* and *BmMETTL14* in a *B. mori* cell line resulted in the arrest of cell cycle progression and deficiency of chromosome alignment and segregation^16^.

*Aedes aegypti* is the most common epidemic mosquito vector in tropical and subtropical regions transmitting a variety of viruses such as dengue virus (DENV), Zika virus (ZIKV), Yellow fever virus, and Chikungunya virus. DENV, a single-stranded positive-sense RNA virus from the viral family *Flaviviridae*^17^, alone is responsible for nearly 400 million annual cases of DENV infectious diseases, including half a million of dengue haemorrhagic fever, from more than 100 countries^18,19^. In mosquitoes, DENV replicates but does not undermine the vector’s survival, which is realized by inhibiting the vector’s immunity to the extent that only allows it to multiply to a non-pathogenic level^20,21^. This entails an optimal balancing between viral replication and host anti-viral responses regulated by the host genes.

Many flaviviruses have m^6^A modification on their RNA in infected mammalian cells. ZIKV RNA is rich in m^6^A modification sites^22,23^, and its replication has been proved to be regulated by methyltransferases METTL3 and METTL14 as well as demethylases ALKBH5 and FTO in human cells. It has been demonstrated that m^6^A can regulate Hepatitis C virus (HCV) infection^22^. Furthermore, researchers analysed the effect of the reading protein YTHDF1-3 on ZIKV replication and found that silencing the m^6^A binding protein, YTHDF2, caused the greatest increase in ZIKV replication^23^. The possible explanation is that YTHDF2 may inhibit the replication of ZIKV by binding to the viral RNA and destabilizing it^23^. Gokhale et al. found that interfering with m^6^A methyltransferases or m^6^A demethylases, respectively, increases or decreases the replication of HCV. During HCV infection, the host cell’s YTHDF protein re-localized to lipid droplets, which are the sites of virus assembly, indicating that YTHDF is able to bind to the virus^22^. The authors also produced mutation of the m^6^A modification site on the *E1* gene of HCV, and the result demonstrated increased binding of viral RNA to the nucleocapsid protein (or core protein) and decreased viral RNA binding to the YTHDF protein, which further led to an increase in infectious virus particles. Gokhale et al., hence, speculated that YTHDF proteins may inhibit the production of infectious virions by competing with nucleocapsid proteins for viral RNA^22^.

In this study, we aimed to investigate the role of m^6^A in DENV replication in the mosquito *Ae. aegypti* by using the Aag2 cell line. Our results suggest that DENV infection indeed leads to changes in m^6^A on mosquito transcripts. We demonstrate that the replication of DENV is positively regulated by the m^6^A methyltransferases and m^6^A binding protein, a YTH family protein. We also identified m^6^A modified sites across the DENV RNA genome. Further, we investigated the role of the highly induced *SUMO-conjugating enzyme Ubc9-B* gene with m^6^A modification in DENV replication. Together, our results shed further light on DENV-mosquito interaction and the role m^6^A modification of host and viral genomic RNA play in the interaction.

## Results

### Identification of RNA m^6^A methylation in *Ae. aegypti*

**Fig. 1:**
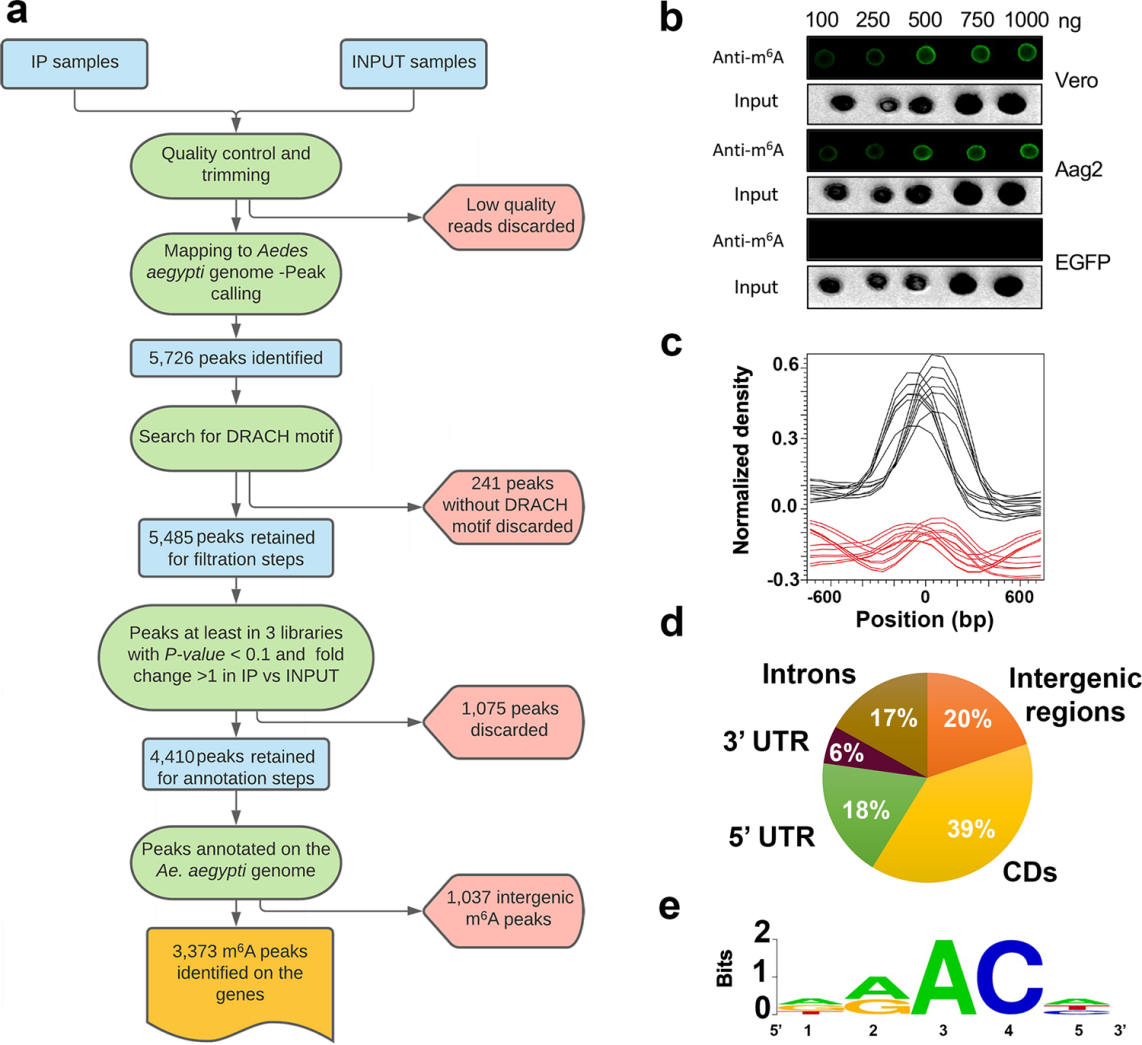
m^6^A modification of *Ae. aegypti* transcripts analysed by dot blot and methylated RNA immunoprecipitation and sequencing (MeRIP-Seq). **a** A diagram showing the MeRIP-Seq data analysis procedure. **b** Confirmation of RNA N^6^-methyladenosine (m^6^A) methylation in *Ae. aegypti*. Total RNA from Aag2 and Vero cells were extracted and subjected to a dot blot assay using a specific anti-m^6^A antibody. EGFP transcripts were synthetised in vitro and used as negative control. The input RNAs were directly stained with ethidium bromide. **c** Normalized density of m^6^A peaks between immunoprecipitated (black lines) and input (red lines) samples following MeRIP-Seq indicating enrichment of m^6^A in the IP samples as part of the quality control of the data. **d** Pie chart showing distribution of m^6^A peaks in *Ae. aegypti* transcripts regions. **e** The consensus m^6^A motif DRA*CH (D = G/A/U, R = G/A, * modified A, H = U/A/C) was enriched in the identified m^6^A peaks.

To find out if m^6^A on RNAs occurs in *Ae. aegypti*, first we performed a dot blot assay by using a commercial monoclonal antibody against m^6^A in an RNA sample isolated from cultured mosquito Aag2 cells. RNA from Vero cells (kidney epithelial cells extracted from an African green monkey) and in vitro synthesized *Enhanced green fluorescent protein* (EGFP) transcripts were used as positive and negative controls, respectively. As shown in Fig. 1b, there are strong m^6^A signals in RNAs from both Aag2 and Vero cells, but not in the *EGFP* transcripts, indicating that the RNAs from *Ae. aegypti* contain m^6^A modification.

To determine the m^6^A landscape and identify detailed m^6^A sites on the *Ae. aegypti* transcripts, we performed methylated RNA immunoprecipitation and sequencing (MeRIP-Seq) on *Ae. aegypti* Aag2 cells (Fig. 1a). Normalized density of m^6^A peaks between immunoprecipitated (IP) and input samples indicated enrichment of m^6^A in the IP samples (Fig. 1c). Following calling peaks in IP over input samples and after all filtering steps, a total of 4410 peaks were identified (Supplementary Data 1).

We then further explored the m^6^A distribution profiles to understand the topological pattern of m^6^A methylation in the mosquito transcriptome. We found that m^6^A peaks were abundant in the coding regions (39%), followed by 20% in the intergenic regions, 18% in the 5’UTR, 17% in introns, and 6% in the 3′ UTRs (Fig. 1d). A snapshot of m^6^A enriched regions on the three chromosomes of *Ae. aegypti* and three representative genes with m^6^A peaks are shown in Supplementary Figs. 1 and 2, respectively. The analysis of m^6^A sequence preference identified AAACU as the most frequent motif (Fig. 1e), showing consistency with the known m^6^A motif DRACH^8–10^. In total, there were 2073 genes identified with m^6^A peaks (some genes have more than one peak).

### Functional annotation of m^6^A modified transcripts from *Ae. aegypti*

**Fig. 2:**
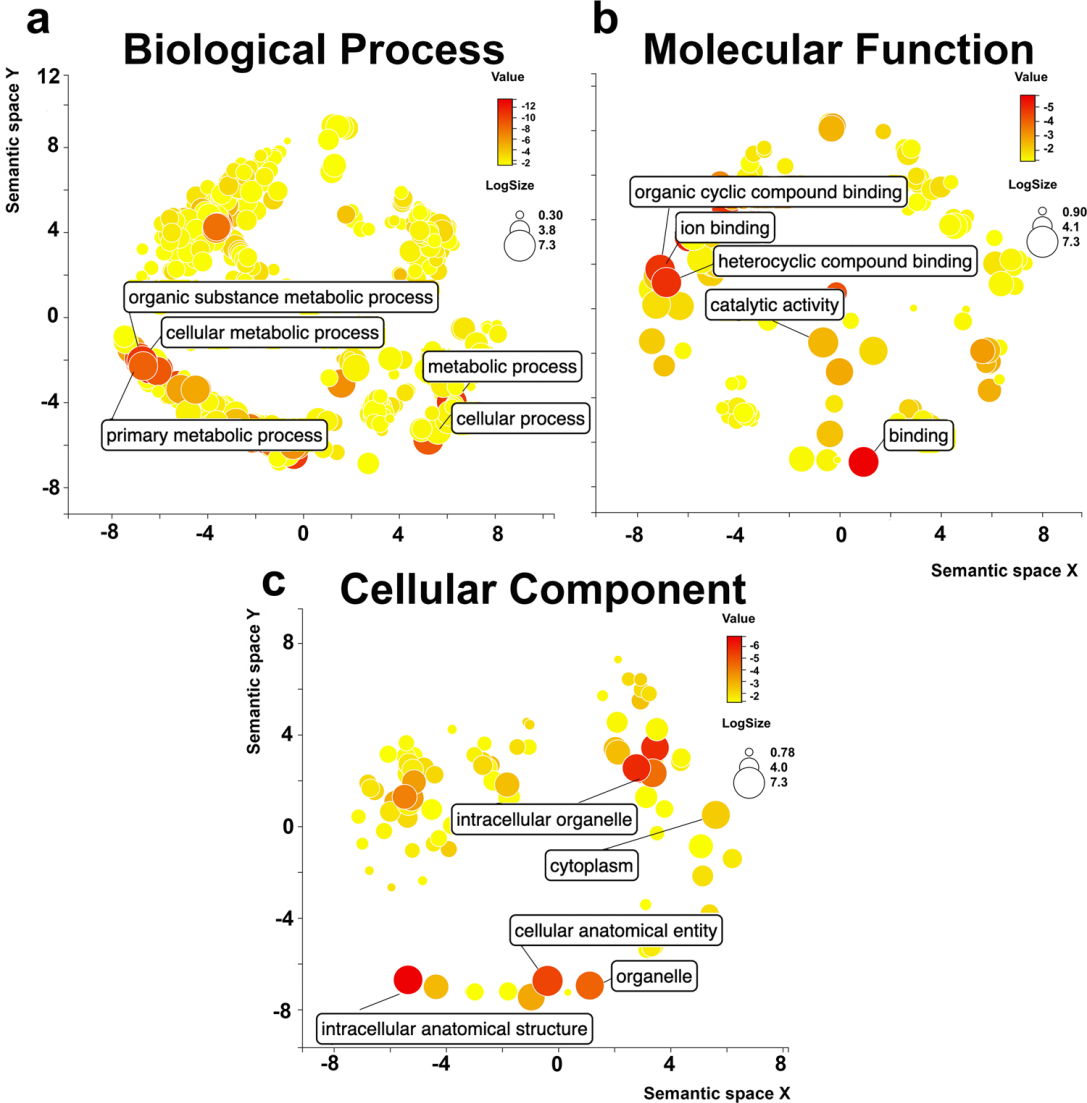
Gene ontology scatterplot of *Ae. aegypti* transcripts with m^6^A modification. The scatterplot shows the cluster representatives of GO terms identified from *Ae. aegypti* transcripts with m^6^A modification generated by Revigo. These GO terms remained after the redundancy reduction, in a two-dimensional space derived by applying multi-dimensional scaling to a matrix of the GO terms’ semantic similarities. **a** Biological process, **b** molecular function and **c** cellular component.

To explore what sort of transcripts are modified by m^6^A in *Ae. aegypti*, we first classified the genes into Gene Ontology (GO) terms. The analysis identified 2502, 903, and 465 GO terms in biological process, molecular function, and cellular components, respectively (Supplementary Data 2). In biological processes category, cellular process, and metabolic process were the top two enriched terms (Supplementary Fig. 3). In molecular function, GO terms associated with “binding” were significantly enriched, and GO terms related to catalytic and hydrolase activity were among highly enriched GO terms. In cellular components category, cellular anatomical entity, intercellular anatomical structure, organelle, ribonucleoprotein complex were among the terms enriched (Supplementary Fig. 3, Supplementary Data 2). The scatterplots generated based on the enriched GO terms in Revigo provide an overview of the most abundant terms in each category (Fig. 2).

### DENV infection changes the m^6^A modification of *Ae. aegypti* cellular transcripts

**Fig. 3:**
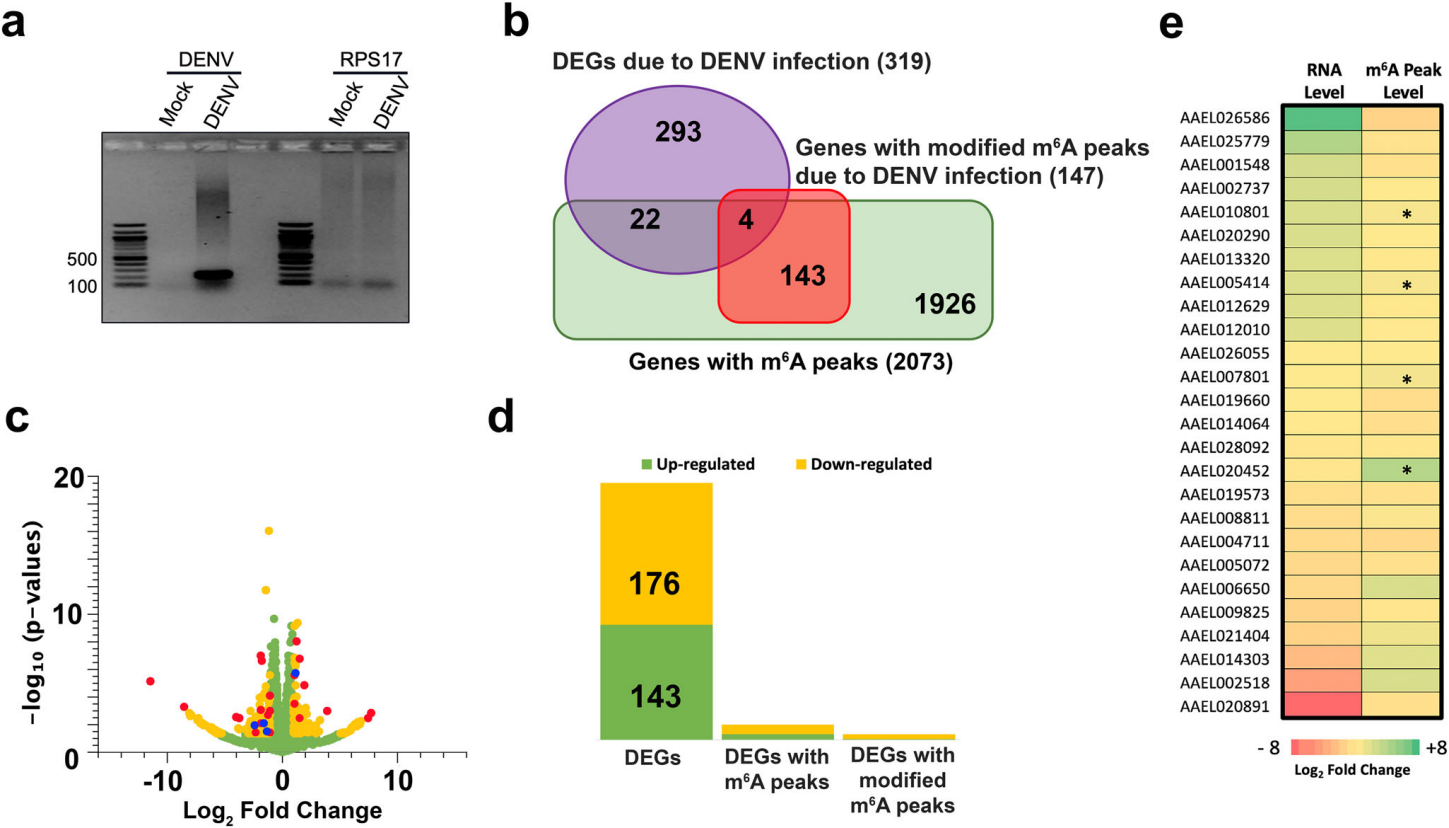
Dengue virus infection alters m^6^A modification of *Ae. aegypti* transcripts. **a** Confirmation of DENV infection of Aag2 cells. RT-PCR analysis of RNA extracted from Aag2 (Mock) and Aag2 cells infected with 1 MOI DENV 5 days after infection using DENV-specific and RPS17 (control) primers. The PCR products were analysed on an agarose gel. **b** Venn diagram showing summarized numbers of differentially expressed genes, with and without m^6^A peak changes. **c** Volcano plot showing differentially expressed genes in Aag2 cells in response to DENV infection. Green and yellow dots represent up-regulated and down-regulated genes with fold change >2 and *p* value <0.05, and those in red and blue are DEGs with m^6^A peaks, with the blue ones being DEGs with changed m^6^A peaks upon DENV infection. **d** Number of significantly differentially expressed genes between mock and DENV-infected Aag2 cells at five days post infection among which there were 26 with m^6^A peaks, of which four showed modified m^6^A upon DENV infection. **e** Heatmap of *Ae. aegypti* genes with m^6^A sites, which were differentially expressed in response to DENV infection (see Table 1 for the list of genes). The four genes that showed differential m^6^A and gene expression upon DENV infection are indicated by asterisks.

To find out whether m^6^A modification alters following infection of mosquito cells with DENV, we compared the m^6^A profile in DENV-infected Aag2 cells (5 days post-infection) with that in uninfected cells by analysing data from MeRIP-Seq. DENV infection was confirmed in the infected samples prior to MeRIP-Seq (Fig. 3a). We identified 147 genes that showed modified m^6^A peaks when mock and DENV-infected samples were compared (Fig. 3b; Supplementary Data 3). Subsequently, we compared the mock and DENV-infected input groups and identified 319 significantly differentially expressed genes (DEGs) after virus infection (Fig. 3b, c; Supplementary Data 4). Of those, 26 genes were found to have m^6^A peaks (Table 1; Fig. 3b, c, blue and red dots). Among these DEGs, there were 176 up-regulated and 143 down-regulated genes, which, respectively, included 10 and 16 genes modified with m^6^A (Fig. 3c–e; Table 1). Of those, m^6^A peaks of nine genes increased and 17 decreased, however, only four genes showed significantly modified m^6^A peak that were also significantly differentially expressed (Fig. 3c; Table 1). The results confirmed that DENV infection did cause changes to m^6^A modification in mosquito cells.

**Table 1:** The list of *Aedes aegypti* genes with m^6^A sites, which were differentially expressed in response to DENV infection.

Name	Gene description	Fold change	*p*-value	DENV-infected (RPKM)	Mock (RPKM)	m^6^A Peak
AAEL026586	Unspecified product	179.5	3.57E–03	0.090	0.000	Peaks 1170, 1171, 1172, 1173, 1174, 1175
AAEL025779	SUMO-conjugating enzyme UBC9-B	10.01	5.28E–03	0.100	0.010	Peak 5676
AAEL001548	Glucosyl/glucuronosyl transferases	2.97	3.88E–03	0.180	0.070	Peak 75
AAEL002737	Cytochrome c oxidase, subunit VIIC, putative	2.92	1.84E–07	2.450	1.090	Peak 419
*AAEL010801*	*Cytochrome b-c1 complex subunit 6*	*2.52*	*4.63E–10*	*3.780*	*1.960*	*Peak 2602*
AAEL020290	60S ribosomal protein L39	2.46	9.30E–09	21.180	11.470	Peak 3632
AAEL013320	Translocon-associated protein, delta subunit	2.21	2.07E–06	2.830	1.760	Peak 2056
*AAEL005414*	*ER membrane protein complex subunit 6*	*2.19*	*1.49E–07*	*3.780*	*2.260*	*Peak 5123*
AAEL012629	Deoxyuridine 5′-triphosphate nucleotidohydrolase	2.11	3.23E–04	1.270	0.780	Peak 1207
AAEL012010	Conserved hypothetical protein	2.1	2.62E–06	1.770	1.100	Peak 3237
AAEL026055	Unspecified product	−2	0.04	0.070	0.180	Peaks 893, 894, 895
*AAEL007801*	*Exonuclease*	*−2.03*	*9.21E–05*	*0.530*	*1.440*	*Peak 2577*
AAEL019660	Zwei Ig domain protein zig-8-like isoform X2	−2.07	1.15E–03	0.070	0.170	Peaks 2648, 2649, 2650
AAEL014064	Glutaredoxin, putative	−2.1	2.62E–06	12.950	35.310	Peak 1640
AAEL028092	Fibroblast growth factor receptor homologue 1	−2.27	2.35E–03	0.060	0.180	Peak 5095
*AAEL020452*	*Titin-like protein*	*−2.44*	*4.00E–02*	*0.005*	*0.020*	*Peaks 249, 250*
AAEL019573	Hemicentin-1	−3.02	8.11E–03	0.020	0.070	Peak 2187
AAEL008811	NADH dehydrogenase 3	−3.36	2.77E–07	2.320	10.690	Peak 5723
AAEL004711	Testis specific leucine rich repeat protein	−3.64	8.32E–03	0.040	0.190	Peak 1555
AAEL005072	MRAS2, putative	−3.84	0.02	0.020	0.080	Peak 5390
AAEL006650	Potassium channel beta	−3.9	6.93E–03	0.009	0.050	Peak 2792
AAEL009825	60S ribosomal protein L13a	−4.83	4.00E–02	0.030	0.210	Peak 5624
AAEL021404	Unspecified product	−5.02	1.00E–02	0.010	0.090	Peak 5605
AAEL014303	Neuroligin	−12.64	3.78E–03	0.002	0.030	Peak 936
AAEL002518	Glutamate receptor, ionotropic kainate 1, 2, 3	−37.12	0.05	0.000	0.010	Peak 4351
AAEL020891	Vacuolar protein sorting-associated protein 13B	−359.05	5.54E–04	0.000	0.080	Peak 5667

Only four genes (in italic) showed differential m^6^A and gene expression upon DENV infection.

### m^6^A machinery is involved in DENV replication in *Ae. aegypti*

**Fig. 4:**
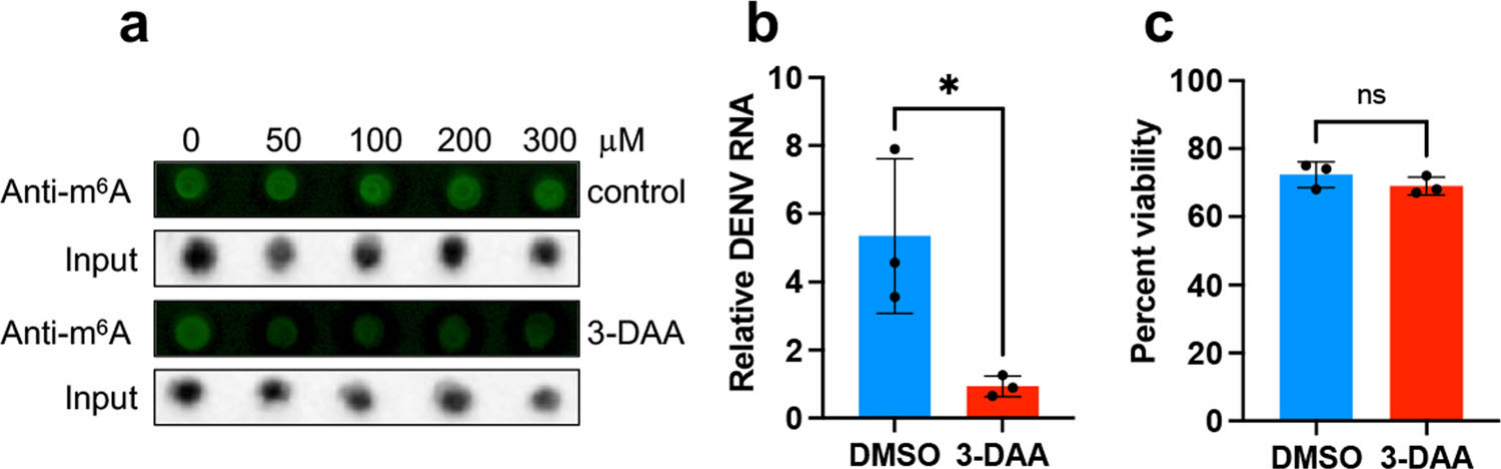
3-Deazaadenosine, an inhibitor of m^6^A, reduces DENV replication in *Ae. aegypti* cells. **a** Confirmation of the effect of 3-DAA on inhibition of m^6^A modification in Aag2 cells. Total RNA from 3-DAA treated Aag2 and control cells (DMSO treated) were extracted and subjected to a dot blot assay using a specific anti-m^6^A antibody. The input RNAs were directly stained with ethidium bromide. **b** Inhibition of DENV replication by 3-DAA. Aag2 cells treated with 100 μM 3-DAA or DMSO were infected with DENV 72 h after treatment with 3-DAA. Cells were collected at 72 hpi for quantification of DENV RNA by RT-qPCR. **c** Viability of Aag2 cells treated with 3-DAA (100 μM) measured at 72 hpi. Error bars represent mean ± S.D. ns, not significant; *p < 0.05, *t*-test.

To find out if overall m^6^A modification has any effect on DENV replication in mosquito Aag2 cells, we used 3-Deazaadenosine (3-DAA; concentration from 0 to 300 μM), a known m^6^A inhibitor^24^. Dot blot results showed 3-DAA could effectively reduce the m^6^A levels of total RNA in Aag2 cells (Fig. 4a). Subsequently, we infected 3-DAA-treated Aag2 cells with 1 MOI DENV. RT-qPCR was performed at 72 hpi. Treatment of cells with 3-DAA significantly decreased (*p* = 0.028) the levels of DENV genomic RNAs (Fig. 4b). Therefore, the results suggest that m^6^A positively regulates the replication of DENV in mosquito cells. Of note, 3-DAA treatment of Aag2 cells had no effect (*p* = 0.279) on cell survival (Fig. 4c).

The homologous genes of the RNA methyltransferases *METTL3* and *METTL14* were blasted against the *Ae. aegypti* genome based on the reported human sequences. We compared the MTA-70 domains from METTL3 and METTL14 between *Homo sapiens* and *Ae. aegypti*. The multiple sequence alignments showed highly conserved domains of MTA-70 between METTL3 and METTL14 proteins from the mosquito and human (Supplementary Fig. 4a). In addition, a homologous gene of the reader *YTHDF3* was found in the *Ae. aegypti* genome. Alignment of YTHDF3 proteins between *Ae. aegypti* and *H. sapiens* showed a low overall degree of protein conservation. However, multiple amino acid sequence alignment showed a highly conserved YTH domain (Supplementary Fig. 4b) between *Ae. aegypti* and *H. sapiens*. Then, we compared these three sequences from eight species to analyse their homologies and established phylogenetic trees by using MEGA5 software with the neighbour-joining method. Accordingly, all the invertebrates (*Drosophila melanogaster, Bombyx mori* and *Ae. aegypti*) clustered together and separated from the vertebrate gene sequences (Supplementary Fig. 5a–c).

**Fig. 5:**
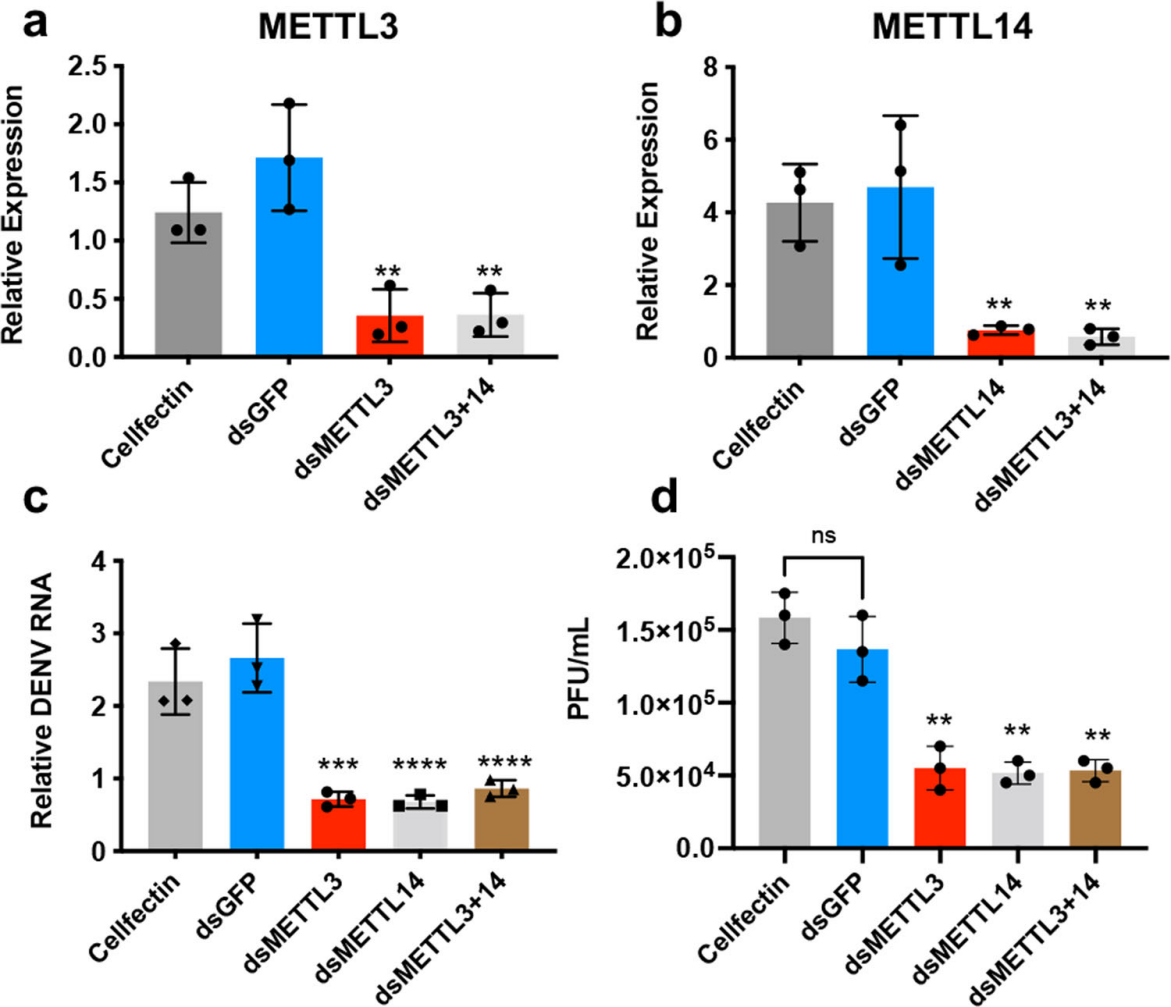
Silencing m^6^A methyltransferase genes reduces DENV replication. Expression levels of **a** *METTL3* and **b** *METTL14* in Aag2 cells transfected with dsRNA to *METTL3* (dsMETTL3), and double transfection with dsRNAs to *METTL3* and *METTL14*. dsRNA to *GFP* (dsGFP) was used as negative control in addition to the Cellfectin transfection reagent only. **c**, **d** Reductions in DENV replication in Aag2 cells by silencing *METTL3*/*METTL14* or both. Cells were collected at 72 hpi for RNA extraction and quantification of DENV RNA by RT-qPCR (**c**), and the supernatants were harvested for quantification of DENV virions by plaque assay (**d**). Error bars represent mean ± S.D. ns, not significant, **p < 0.01; ***p < 0.001; ****p < 0.0001. ANOVA test with post hoc comparisons.

To further determine whether m^6^A modification affects DENV replication, we knocked down the m^6^A methyltransferases, *METTL3* and *METTL14*, in Aag2 cells through RNAi and infected the cells with DENV. RT-qPCR was performed on RNA extracted from cells collected at 72 h post infection (hpi), which confirmed effective silencing of *METTL3* (75%) and *METTL14* (85%) (Fig. 5a, b). *METTL3* and *METTL14* depletion significantly decreased (∼2.5-4-fold; *p* < 0.0001) the levels of DENV genomic RNA (gRNA; Fig. 5c). Silencing of *METTL3* and *METTL14* together also led to declined DENV gRNA levels but to the same extent as single gene silencing (Fig. 5c). *METTL3* and *METTL14* depletion, separately or together, also decreased (*p* < 0.0001) the titre of DENV determined by plaque assay (Fig. 5d). These results suggested a positive correlation between the m^6^A writers and DENV replication.

**Fig. 6:**
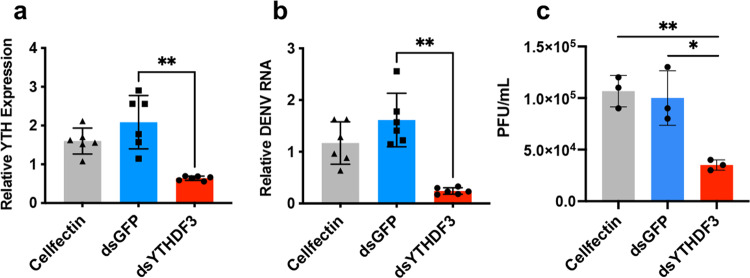
m^6^A YTHDF3 reader modulates DENV replication in *Ae. aegypti* cells. **a** Confirmation of silencing of *YTHDF3* homologue by dsRNA (dsYTHDF3) in Aag2 cells. dsRNA to *GFP* (dsGFP) was used as a negative control in addition to Cellfectin transfection reagent only. **b**, **c** Inhibition of DENV replication by *YTHDF3* silencing. Aag2 cells transfected with dsRNA targeting *GFP* or dsRNA targeting *YTHDF3* were infected with DENV 72 h after transfection. Cells were collected at 72 hpi for RNA extraction and quantification of DENV RNA by RT-qPCR (**b**), and the supernatants were harvested for quantification of DENV virions by plaque assay (**c**). Error bars represent mean ± S.D. *p < 0.05; **p < 0.01. ANOVA test with post hoc comparisons.

As for the effect of m^6^A reader on DENV replication, we knocked down *YTHDF3* by using dsRNA to the gene (Fig. 6a). RT-qPCR analysis was performed at 72 hpi. Figure 6b shows that *YTHDF3* depletion significantly reduced (*p* < 0.001) the levels of DENV gRNA. Also, *YTHDF3* depletion led to decreased (*p* < 0.005) titre of DENV (Fig. 6c).

### *Ubc9* is m^6^A modified and facilitates replication of DENV

**Fig. 7:**
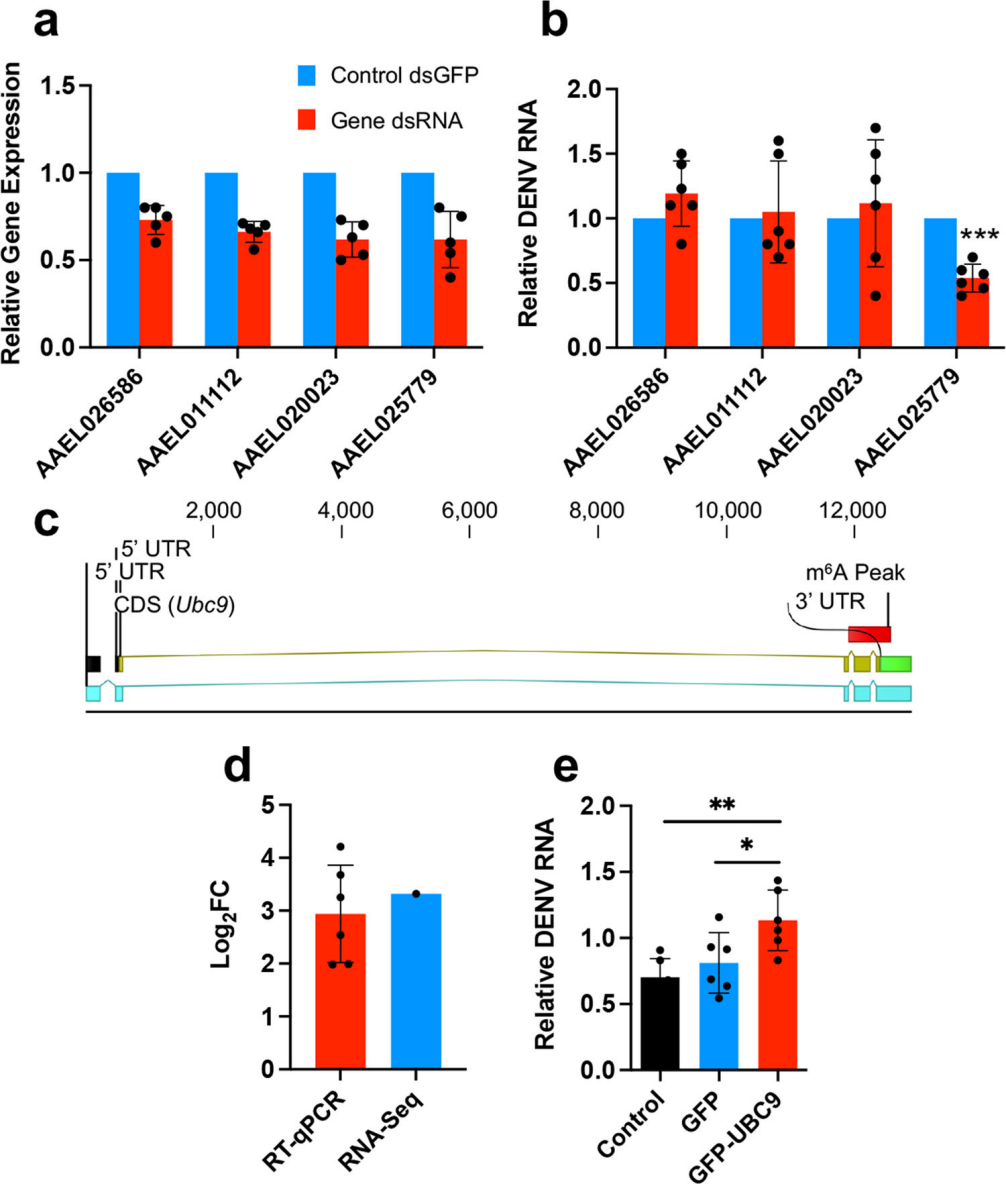
*Ubc9* facilitates DENV replication in *Ae. aegypti* cells. **a** Confirmation of silencing of four genes selected from differentially expressed genes by RNAi in Aag2 cells. **b** Inhibition of DENV replication by *Ubc9* (AAEL025779) silencing. Aag2 cells transfected with dsRNA targeting *GFP* or dsRNA targeting the four genes were infected with DENV-2 72 h after transfection. Cells were collected at 72 hpi for quantification of DENV RNA by RT-qPCR. Control *GFP* expression levels in **a** and **b** were adjusted to 1. ***p < 0.001 (*t*-test). **c** Location of the m^6^A peak in *Ubc9*. **d** RT-qPCR analysis of RNA from Aag2 cells infected with DENV for 72 h showed consistency in up-regulation of *Ubc9* transcript levels with the RNA-Seq data. FC, fold change. **e** Aag2 cells transfected with pSLfa-GFP or pSLfa-GFP-Ubc9 were infected with DENV 72 h after transfection. Cells were collected at 72 hpi for RNA extraction and quantification of DENV genomic RNA by RT-qPCR. Error bars represent mean ± S.D. *p < 0.05; **p < 0.01. One-way ANOVA with post hoc comparisons.

We selected four most significantly DEGs (AAEL026586, AAEL011112, AAEL020023, AAEL025779, coding, respectively, for an unspecified protein, alcohol dehydrogenase, histone H2B, and Ubc9; Supplementary Data 4) and explored their potential influence on DENV replication. Two of these genes (AAEL026586 and AAEL025779) were m^6^A modified and two were not (AAEL011112 and AAEL020023). As these DEGs genes were all up-regulated, we, respectively, knocked them down in Aag2 cells (Fig. 7a) and then infected them with DENV-2 at MOI of 1. The results indicated that depletion of AAEL025779 gene reduced DENV replication (*p* = 0.0007), but depletion of the other three genes did not bring significant changes to DENV replication (Fig. 7b). AAEL025779 encodes the SUMO-conjugating enzyme Ubc9, an important enzyme in the SUMOylation pathway, and was found to be m^6^A modified at the 3′UTR (Fig. 7c). Up-regulation of *Ubc9* was also confirmed by RT-qPCR in Aag2 cells infected with DENV for 72 h, consistent with the RNA-Seq data (Fig. 7d).

Subsequently, we constructed a plasmid, pSLfa-Ubc9-GFP, to overexpress *Ubc9* in fusion with *GFP* in Aag2 cells and then infected the cells with DENV-2 at MOI of 1. The results showed that the overexpression of Ubc9 led to increased replication of DENV (Fig. 7e). Overall, the results suggest that Ubc9 facilitates the replication of DENV.

### Inhibition of m^6^A modification decreases the transcript levels of *Ubc9*

**Fig. 8:**
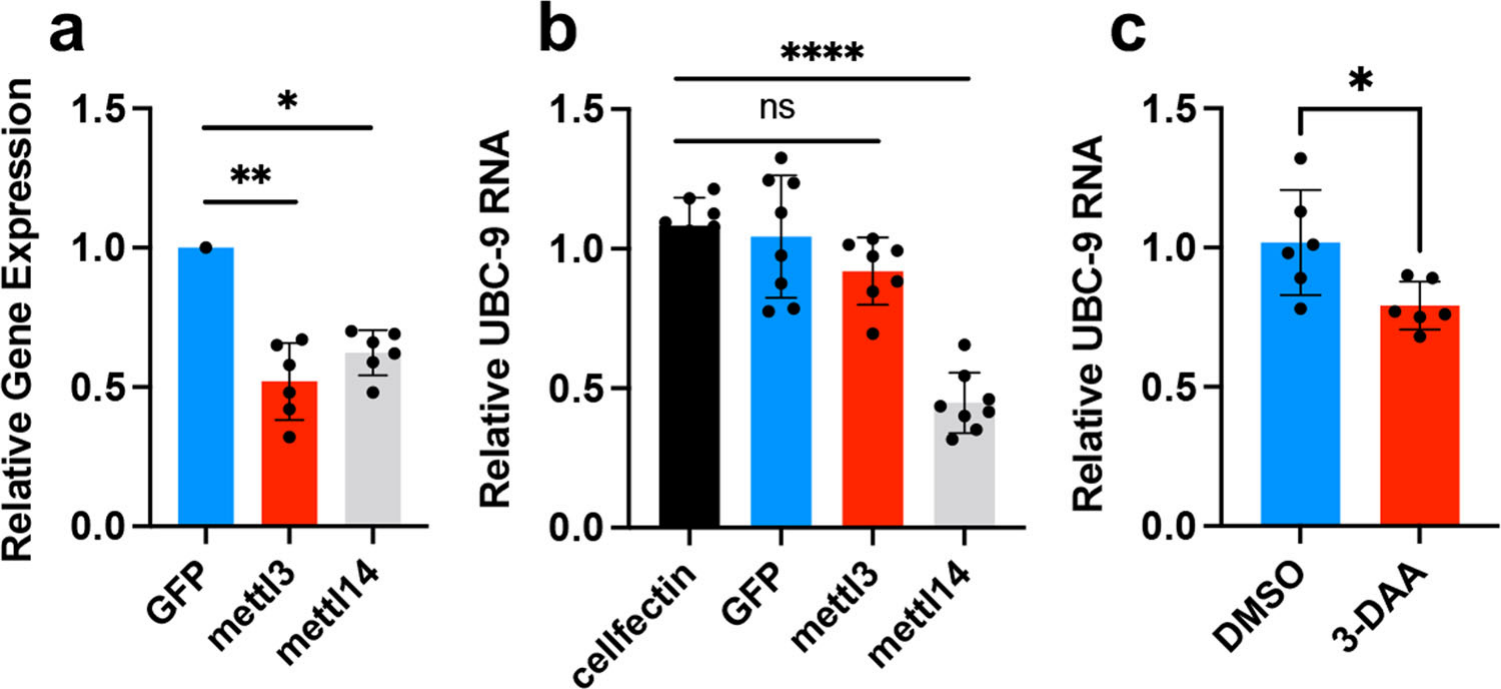
Suppression of m^6^A modification reduces *Ubc9* transcript levels. **a** Confirmation of silencing of *METTL3* and *METTL14* by RNAi in Aag2 cells. Control GFP was adjusted to one. *p < 0.05; **p < 0.01 (one-way ANOVA with post hoc comparisons). **b** Decrease of *Ubc9* expression by silencing m^6^A writers. Aag2 cells were transfected with dsRNA targeting *GFP* or dsRNA targeting *METTL3* or *METTL14*. Cells were collected at 72 days post-transfection for quantification of *Ubc9* RNA levels by RT-qPCR. ****p < 0.0001; ns, not significant, one-way ANOVA with post hoc comparisons. **c** RT-qPCR was used to evaluate the transcript levels of *Ubc9* after using m^6^A inhibitor, 3-DAA. *p < 0.05 (*t*-test). Error bars represent mean ± S.D.

In order to explore the role of m^6^A modification in *Ubc9* expression, we knocked down the m^6^A methyltransferases, *METTL3* and *METTL14*, separately through RNAi and infected the cells with DENV. RT-qPCR was performed at 72 h post infection (hpi). Figure 8a shows that the dsRNA of *METTL3* and *METTL14* reduced the transcript levels of the two m^6^A methyltransferases by about 50% and 40%, respectively. While depletion of *METTL14* significantly decreased the levels of *Ubc9* RNAs (*p* < 0.0001), depletion of *METTL3* did not lead to a statistically significant reduction of *Ubc9* transcript levels (Fig. 8b). In addition, we inhibited m^6^A modification in Aag2 cells by using the known m^6^A inhibitor, 3-DAA. As a result, the transcript levels of *Ubc9* significantly decreased (Fig. 8c; *p* = 0.0233). Taken these results, we found that the expression of *Ubc9* could be regulated by m^6^A modification.

### m^6^A modification affects the mRNA stability of *Ubc9*

**Fig. 9:**
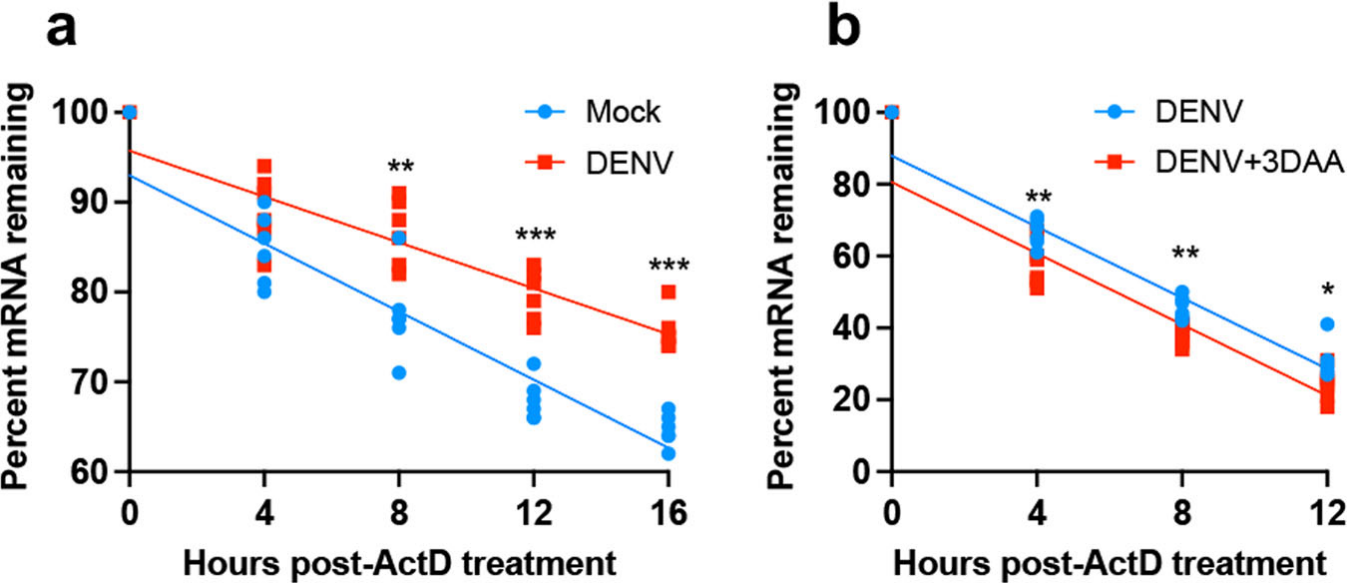
DENV infection promotes *Ubc9* RNA stability. **a** Measurement of *Ubc9* RNA in mock and DENV-infected Aag2 cells. At 72 hpi, cell culture medium was replaced with medium containing 1 μM ActD. RNA was collected at the indicated times post-treatment and subjected to RT-qPCR to determine remaining relative RNA levels. **p < 0.01; ***p < 0.001 (*t*-test). **b** Measurement of *Ubc9* RNA in m^6^A DENV-infected m^6^A-inhibited Aag2 cells with 3-DAA. At 72 hpi, cell culture medium was replaced with medium containing 3-DAA. After 24 h post treatment, cell culture medium was replaced with medium containing ActD. RT-qPCR was used to measure the remaining relative RNA levels *p < 0.05; **p < 0.01 (*t*-test).

m^6^A can alter mRNA metabolism, including splicing, translation, and stability to affect gene expression^2^. For the specific gene *Ubc9*, we took a closer look at its mRNA stability, and explored whether m^6^A modification affects the expression of *Ubc9* through regulating its mRNA stability. We used Actinomycin D, an inhibitor of transcription, to assess the mRNA stability of *Ubc9* during DENV infection and found that upon DENV infection the stability of *Ubc9* mRNA increased (Fig. 9a). In addition, 3-DAA was used to inhibit m^6^A modification in DENV-infected Aag2 cells, and then the *Ubc9* mRNA stability was assessed (Fig. 9b). The results showed that inhibiting m^6^A methylation decreased the mRNA stability of *Ubc9*.

### m^6^A methylation in DENV RNA

**Fig. 10:**
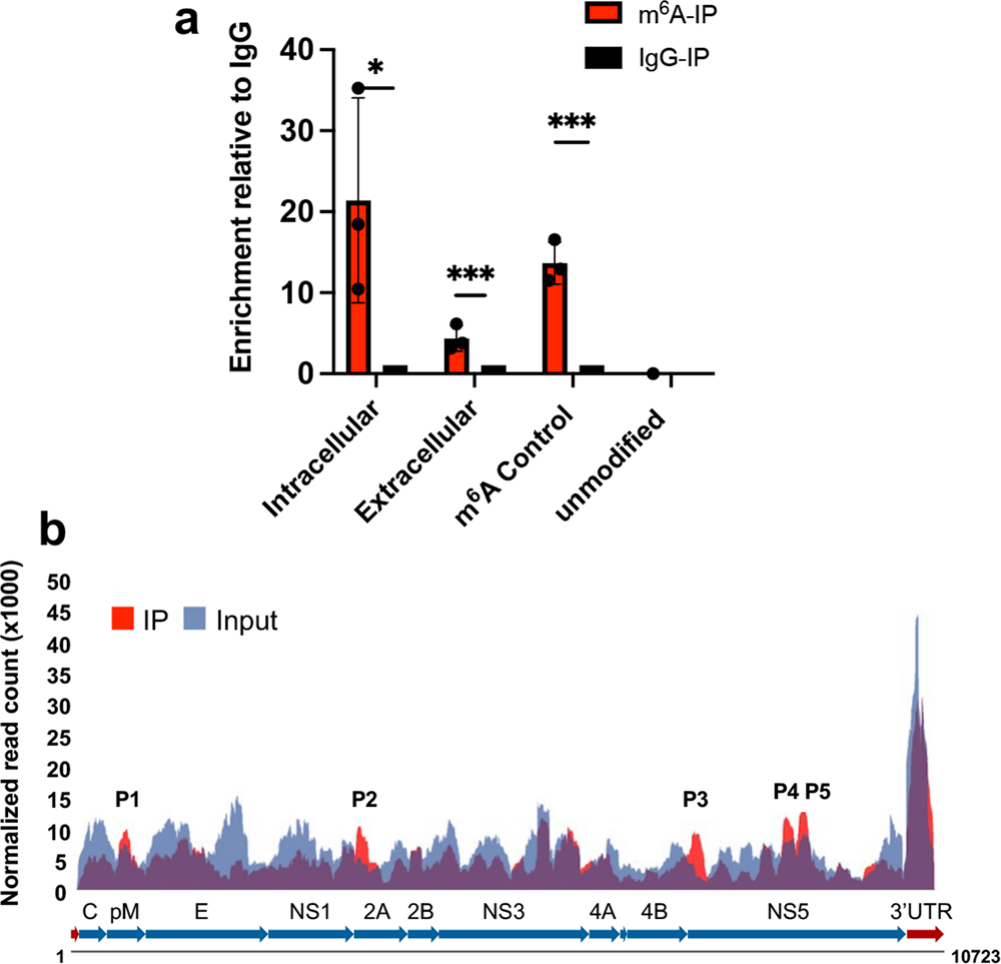
DENV RNA is modified by m^6^A in infected mosquito cells. **a** MeRIP-RT-qPCR analysis of RNA harvested from DENV-infected Aag2 cells (72 hpi) and immunoprecipitated with anti-m^6^A or IgG using primers to DENV genome. For the extracellular virions, the supernatant collected from DENV-infected cells was treated at room temperature with 17 μg of RNase A per ml for 20 min to remove any naked RNA. Eluted RNA was quantified as a percentage of input. m^6^A Control, positive control RNA with m^6^A modification; unmodified, negative control RNA without m^6^A modification. Error bars represent mean ± S.D. *p ≤ 0.01, ***p ≤ 0.001 (*t*-test). **b** Map of m^6^A-binding sites in the DENV RNA genome by MeRIP-Seq of RNA isolated from DENV-infected Aag2 cells based on three biological replicates. Read coverage, normalized to the total number of reads mapping to the viral genome for each experiment, is in red for MeRIP-Seq and in blue for input RNA-Seq. The five identified peaks (P1-P5) are indicated on the graph.

It has been reported that DENV genomes contain m^6^A modification in infected mammalian cells^22^. We hypothesized that the DENV gRNA is also modified by m^6^A during infection in infected mosquito cells. To confirm this, we carried out MeRIP-RT-qPCR on total RNA harvested from DENV-infected Aag2 cells collected at 72 hpi. This involved m^6^A RNA enrichment followed by RT-qPCR using primers to the DENV genome. DENV RNA was specifically enriched by the anti-m^6^A antibody, but not by IgG (Fig. 10a). This enrichment was found in DENV RNA from both intracellular as well as extracellular fractions, suggesting that the released virions most likely also contain m^6^A modified gRNA.

We next mapped the sites of the DENV RNA genome modified by m^6^A using the MeRIP-Seq data. We identified five m^6^A peaks in DENV gRNA (Fig. 10b and Supplementary Table 1). This further confirmed that DENV gRNA is modified by m^6^A in infected mosquito cells. Two m^6^A peaks were found on the NS5 coding region and one peak each on the pM, NS1-NS2A and NS4B-NS5 coding regions. DENV pM and NS1 facilitate the formation and maturation of the viral particles. NS2A and NS4B are involved in DENV virion assembly. NS5 contains a methyltransferase and a polymerase domain, which are essential for the replication of the DENV RNA genome.

## Discussion

In this study, we demonstrated m^6^A modification of *Ae. aegypti* RNAs and further determined the global landscape of m^6^A modifications on the mosquito transcriptome through MeRIP-Seq. In total, we identified 4410 m^6^A-related peaks that were mostly in the coding region followed by the intergenic regions. Analysis of the sequencing results shed light on the function of the m^6^A modified transcripts. These were classified in the three GO categories of biological processes, molecular functions, and cellular components, with most of them found in biological processes.

Previous studies have demonstrated alterations in the m^6^A profile of mammalian cells upon virus infection^22,23,25,26^. Relevant to this study, several viruses from *Flaviviridae* (DENV, ZIKV, WNV and HCV) were shown to change the landscape of m^6^A on the transcriptome of human Huh7 cells^27^. Further, ZIKV infection led to changes in m^6^A modification of transcripts in human embryonic kidney 293T cells^23^. Gokhale et al. discovered that DENV infection changed the m^6^A modification of *RIOK3* and *CIRBP* genes, through interferon-β (IFN) and the endoplasmic reticulum stress pathway. They further demonstrated that the alteration of the m^6^A modification on RIOK3 and CIRBP can, in return, enhance DENV replication in human cells^27^. Given this, it is possible that DENV utilizes these pathways to change the expression of the host pro-viral and anti-viral genes (*RIOK3* and *CIRBP*) and as a result benefit its replication^27^. However, we did not find any changes either in the expression of the *RIOK3* and *CIRBP* genes or in their m^6^A modification in mosquito Aag2 cells. In addition, we sought out the human genes showing significant m^6^A peak changes upon DENV infection from Gokhale et al. and found their homologues in the *Ae. aegypti* genome to see if any of them show changes in m^6^A during DENV infection^27^. Yet none of them, including the homologues of *RIOK3* and *CIRBP*, showed m^6^A changes. This suggests that DENV infection may have different effects on m^6^A modification between humans and mosquitoes. It is known that DENV presents different pathogenicities in the mosquito vector and the human host and this could be reflected in differences in m^6^A modifications in the host and the vector. In the mosquito vector, DENV presents rather a persistent infection, whereas the virus infection can be acute in humans^28,29^.

The m^6^A machinery functions through the regulation of three groups of enzymic proteins in host cells, including the m^6^A writers, readers, and erasers^7^. A large body of research evidence has confirmed that depletion of these proteins affects viral replication. Interfering with the expression of the m^6^A reader, YTHDF1-3, enhanced the production of ZIKV and HCV through reducing the binding of the reader to the viral genomes where m^6^A modifications occur^22,23^. In addition, some host RNAs which contain m^6^A sites have been reported to affect viral replication. For example, methylation of oxoglutarate dehydrogenase (OGDH) mRNA was shown to decrease the replication of Vesicular stomatitis virus (VSV). The outcome provided a novel strategy for researchers to develop a drug targeting the methylation of OGDH mRNA for the control of VSV infection^25^. In DENV infection, we found the mosquito m^6^A writer METTL3, METTL14 and m^6^A reader YTH family protein affect DENV replication. Silencing of these genes through RNAi led to reduced DENV replication suggesting that unlike in human cells, m^6^A machinery facilitates DENV replication. However, the off-target effects of these RNAi experiments done in previous works and this study cannot be ruled out.

m^6^A modification on viral genomic RNA can affect viral infection. The m^6^A sites on the envelope gene of HCV and ZIKV have been confirmed to facilitate suppression of viral replication through binding with the reader, YTHDF protein^22,23^. In the 3′UTR of DENV, there are two stem-loops whose function is to control viral transmission from mosquito to human^30^. Of note, while we did not find any m^6^A peak in DENV 3′UTR replicated in mosquito cells, one peak was detected in this region (10273-10300) on the DENV genome when replicated in mammalian cells^22^. As such, the m^6^A modification on the 3′UTR may be related to the vector-borne transmission mechanism^31^. We found that the genomic RNA of DENV contains m^6^A modification in precursor membrane (pM), non-structural protein 2A (NS2A), and non-structural protein 5 (NS5) coding regions when infecting mosquito cells. However, the function of m^6^A sites on the DENV RNA genome remains to be investigated. Gokhale et al. found DENV genome had prominent m^6^A sites in 11 sites in infected human Huh7 cells^27^. Of those, three peaks that were in the NS5 coding region have overlaps with those we found in the DENV genome replicated in Aag2 mosquito cells. This is a possible demonstration that some of the m^6^A modification sites on the viral genome may be conserved when DENV replicates in human and mosquito cells.

The present study identified 26 significantly DEGs with m^6^A peaks, some with m^6^A changes, when mock and DENV infected samples were compared. An altered gene *Ubc9*, which is m^6^A modified in mosquito cells, was found to facilitate DENV replication as its silencing led to reduced virus replication. Previous reports have indicated that several viruses may hijack the Ubc9 protein to support their infection^32–34^. Our results suggest that presumably DENV increases the transcript levels of *Ubc9* by increasing their stability mediated by m^6^A modification. The *Ubc9* gene encodes an important enzyme known as SUMO-conjugating enzyme Ubc9, which is involved in the SUMOylating pathway^35^. Small ubiquitin-like modifier (SUMO) proteins are involved in SUMOylation, a posttranslational modification process that is reversible and does not lead to protein degradation, unlike ubiquitination. SUMOylation affects various cellular processes, such as transcriptional regulation, apoptosis, protein stability, nuclear-cytosolic transport, cell cycle and virus infection^36^. Replication of a wide variety of viruses can be facilitated through impaired SUMOylating processes. Decreased SUMOylation of anti-viral proteins can interrupt anti-viral immune responses. On the other hand, some viruses may benefit from up-regulated SUMO proteins. For example, Influenza virus infection can cause a global increase in cellular SUMOylating and as a result enhanced replication of the virus^37^. The Ubc9 protein has been widely studied as a target of several viruses, including DENV, ZIKA, and HIV in mammalian cells. It has been shown that human Ubc9 protein can interact with HIV Gag protein and enhance replication of HIV^38^. In addition, Ubc9 has been reported to interact with DENV proteins, such as the DENV-2 envelope and NS5 proteins^39,40^. Silencing of *Ubc9* gene led to reduced DENV-2 replication in human Huh7 cells, consistent with our results in mosquito cells^40^. As such, we presume that the SUMOylation pathway may be involved in DENV infection in mosquitoes, in association with alteration in m^6^A modification. In contrast, it was recently shown that depletion of core SUMOlyation effector proteins, including Ubc9, in *Ae. aegypti* AF5 cells led to enhanced replication of ZIKV, Semliki Forest virus, and Bunyamwera virus^41^.

There are only few studies that have established a link between m^6^A and SUMOylation with Ubc9 involvement. In human 293T cells, it was shown that METTL3 is SUMOylated and its SUMOylation reduces its m^6^A methyltransferase activity^42^. Further, in human liver cancer cell line MHCC97H, it was also found that METTL3 is SUMOylated by Ubc9, and increase in SUMOylation of METTL3 correlated with Ubc9 up-regulation^43^. Overall, up-regulation of *Ubc9* expression in mosquito Aag2 cells following DENV infection, through an increase in the stability of its mRNAs mediated by m^6^A modification, may lead to increases in SUMOylation, which is known to facilitate DENV replication in human cells^40^. However, establishing any direct link between m^6^A and SUMOylation in mosquito cells requires further investigations.

In summary, our results indicate that m^6^A RNA modification occurs in *Ae. aegypti*, and DENV infection alters the m^6^A repertoire of mosquito transcripts. The alteration of m^6^A caused by DENV in mosquitoes is, however, different from that found in human transcripts. Furthermore, we found the role of m^6^A machinery in positively affecting the replication of DENV. We also identified m^6^A modifications of the DENV genome. Furthermore, the transcript levels of a SUMOylation core protein, Ubc9, which is m^6^A modified, were found to increase with DENV infection. The results reveal that DENV may increase the stability of *Ubc9* transcripts ultimately supporting its own replication. The link and possible crosstalk between m^6^A modification and SUMOylation could be important in regulation of DENV replication, which deserves further exploration in future studies. This work contributes to our understanding of the differences of DENV m^6^A modification between human and *Ae. aegypti* infection, and the role of epigenetics in gene regulation during DENV infection in the mosquitoes.

## Methods

### Cells and viruses

*Ae. aegypti* Aag2 cells were cultured in 1:1 Mitsuhashi-Maramorosch and Schneider’s insect medium (Invitrogen) supplemented with 10% foetal bovine serum (Bovogen Biologicals) at 28 °C. Vero cells were grown in Opti-MEM medium supplemented with 2% foetal bovine serum in a 37 °C incubator with CO_2_. DENV serotype 2 East Timor strain (DENV-2 ET300) was used in infection experiments.

For virus infections, Aag2 cells were seeded in 6-well plates and allowed to attach for 1 h. Cells were then infected with 1 multiplicity of infection (1 MOI) of DENV-2 for 1 h at room temperature. Subsequently, fresh medium was added to cells and incubated at 28 °C. Duration of infections is indicated in the text or figure legends.

### Virus titration

Plaque assay was used to measure virus titre in media as described previously^44^. Briefly, Vero cells (4 × 10^4^ in 50 μl medium) were seeded in a 96-well plate and incubated at 37 °C until 90–100% confluent. Then, collected medium containing virus was diluted to 10^0^, 10^−1^, 10^−2^, 10^−3^ with medium, and added into wells. Plates were first incubated for 1 h with shaking at room temperature and then incubated for an additional hour at 37 °C. After that, medium was removed from each well and to each well 50 μl of a mixture of 50% CMC gel (3% carboxymethyl cellulose) + 50% medium with FBS (final concentration of 4%) was added. The plate was incubated at 37 °C for 72 h. The overlay was discarded, then cells were fixed by adding 50 μl of ice-cold 80% acetone in PBS at −20 °C for 20 min and then air-dried overnight. Five percent skim milk in PBST (1× phosphate-Buffered Saline, 0.1% Tween) was used to block the cells. Cells were incubated with the primary antibody against DENV-2 Envelope protein (human) in 1:1000 dilution in 0.1% skim milk in 1×PBST for 2 h at 37 °C. After that, the plate was washed three times with PBS containing 0.05% Tween 20. Subsequently, cells were incubated with a secondary IR Dye conjugated anti-human antibody (Sigma) with 1% skim milk in PBST (1:5000) at 37 °C for 1 h. The plate was washed three times with PBS containing 0.05% Tween 20 and dried for 0.5 h. Virus plaques were detected by LI-COR Biosciences Odyssey infrared Imaging System and application software.

### Dot blot

Total RNA was isolated from Aag2 and Vero cells. RNAs of the *EGFP* gene were synthesized in vitro by T7 RNA polymerase. Samples were diluted to 100 ng/μl and spotted on a polyvinylidene fluoride membrane (PVDF, Roche, Indianapolis, IN, USA). The membrane was UV-cross-linked and air-dried. The membrane was then blocked in the blocking buffer (5% skim milk in PBST) for 2 h at room temperature, and incubated with an m^6^A monoclonal antibody (1:1000; Synaptic Systems) overnight at 4 °C. After three washes with wash buffer (5% skim milk in PBST) for 10 min each, the membrane was incubated with an anti-mouse secondary antibody conjugated with an IR Dye (1:5000; Sigma) for 60 min at room temperature. After three washes as above, signals were detected with LI-COR Biosciences Odyssey infrared Imaging System and application software.

### MeRIP-Seq

MeRIP-Seq was carried out by combining and modifying approaches described previously^16,27^. Aag2 cells seeded in 6-wells plates were infected with DENV serotype 2 East Timor strain (DENV-2 ET300) (MOI 1). At 5 days post-infection, total RNA was extracted using QIAzol lysis reagent (QIAGEN) and treated with TURBO DNase I (Thermo Fisher). Total RNA was fragmented using the RNA Fragmentation Reagent (Thermo Fisher) for 15 min. MeRIP was performed using EpiMark N^6^-methyladenosine Enrichment kit (NEB). Twenty-five microlitres of Protein G Dynabeads (Thermo Fisher) per sample was washed three times in MeRIP buffer (150 mM NaCl, 10 mM Tris-HCl [pH 7.5], 0.1% NP-40), and incubated with 1 μL anti-m^6^A antibody (Synaptic System) for 2 h at 4 °C with rotation. After washing three times with MeRIP buffer, anti-m^6^A conjugated beads were incubated with purified mRNAs with rotation at 4 °C overnight in 300 μL MeRIP buffer with 1 μL RNase inhibitor (recombinant RNasin; Promega). Ten percent of the mRNA sample was saved as the input fraction. Beads were then washed twice with 500 μL MeRIP buffer, twice with low salt wash buffer (50 mM NaCl, 10 mM Tris-HCl [pH 7.5], 0.1% NP-40), twice with high salt wash buffer (500 mM NaCl, 10 mM Tris-HCl [pH 7.5], 0.1% NP-40), and once again with MeRIP buffer. m^6^A-modified RNAs were eluted twice in 100 μL of MeRIP buffer containing 5 mM m^6^A salt (Santa Cruz Biotechnology) for 30 min at 4 °C with rotation. Eluates were pooled and concentrated by ethanol precipitation. RNA samples were submitted to Genewiz for RNA sequencing with Illumina Hiseq PE150. Three replicates were submitted per sample (IP and input), totalling 12 samples.

### RNA-Seq data analysis

The CLC Genomics Workbench version 20.0.4 was used for bioinformatics analyses in this study. All input and m^6^A immunoprecipitated RNA (IP) libraries were trimmed from sequencing primers, adaptor sequences and low-quality reads. Low-quality reads (quality score below 0.05) and reads with more than two ambiguous nucleotides were discarded. Clean reads were aligned to the latest version of the *Ae. aegypti* reference genome (GCA_002204515.1) with mismatch, insertion, and deletion cost of 2, 3, and 3, respectively. Mapping was performed with stringent criteria and allowed a length fraction of 0.8 in mapping parameter, which encounter at least 80% of nucleotides in a read must be aligned to the reference genome. The minimum similarity between the aligned region of the read and the reference sequence was set at 80%.

The peak calling process was conducted by using CLC Epigenomics toolbox to identify the enriched regions of m^6^A peaks by comparing reads from the IP and input samples. As the first step of quality control, the input data was examined to check if the data satisfy the assumptions made by the algorithm and to compute several quality measures such as relative strand correlation and normalized strand coefficient^45–47^. After passing the quality control, estimated fragment length and estimated window size were calculated for peak calling process^48^. The CLC shape-based peak caller uses the characteristic of peak shape and enriched read coverage to identify peaks in MeRIP-seq data. The peak shape filter was applied to the experimental data and a score and the *p-*value were calculated at each genomic position. The score was obtained by extracting the genomic coverage profile of a window centred at the genomic position and then comparing this profile to the peak shape filter. The result of this comparison was defined as peak shape score by CLC Genomic Workbench. Once the peak shape score for the complete genome was calculated, peaks were identified as genomic regions where the maximum peak shape score was >1.28 and the *p*-value is smaller than the defined threshold (0.1). The centre of the peak was then identified as the genomic region with the highest peak shape score, and the boundaries were determined by the genomic positions where the peak shape score became negative^48^. The peak regions were extracted, and we searched for the presence of DRACH motif as methylation site within both strands of the peak regions. The peaks without DRACH motif enrichment were discarded. Peaks considered as true m^6^A peaks, if they were detected in at least two libraries with more than five associated reads in each individual IP samples and their normalised fold change in IP vs Input samples was greater than one (Fig. 1a). To define genes associated with the m^6^A peaks, the reference genome was annotated by the identified peaks and their relative positions data were exported if it overlapped with any annotated gene region. The peaks and the associated genes were then manually checked and any peaks within the gene region were retained for further downstream analysis.

We performed the RNA-Seq analysis to identify the involvement of m^6^A sites in host dysregulated genes in response to DENV infection. We mapped the input reads to the *Ae. aegypti* reference genome and their expression profiles were produced by distributing and counting the reads across genes and transcripts. The relative expression levels were produced as RPKM (reads per kilobase of exon model per million mapped reads) values. We also used the same tools to identify altered m^6^A peaks in response to DENV infection. We used the normalised TPM (transcripts per million) value to compare m^6^A site modification in DENV-infected and non-infected samples. For both m^6^A peaks and DEGs, the total read counts were calculated based on average of total mappable reads from three replicate libraries.

The multi-factorial statistics based on a negative binomial generalized linear model (GLM) was used to generate differential expression profile. Each gene is modelled by a separate GLM and this approach allows us to fit curves to expression values without assuming that the error on the values is normally distributed. TMM (trimmed mean of M values) normalization method was applied to all datasets to calculate effective library sizes, which were then used as part of the per-sample normalization^49^. The Wald Test was also used to compare each sample against its control group to test whether a given coefficient is non-zero. We considered genes with more than 2-fold change and *p*-value of <0.05 as statistically significantly modulated genes.

All the *Ae. aegypti* genes with m^6^A peaks were uploaded on the Blast2GO^50^ server for functional annotation and GO analysis. We used BLAST, InterProScan^51^, enzyme classification codes (EC) and EggNOG^52^ to determine the GO terms of the differentially expressed sequences. More abundant terms were computed for each category of molecular function, biological process, and cellular components. An enrichment analysis using Fisher’s Exact Test was done using all AaegL5.0 annotated genes as the reference dataset by FatiGO package, which is integrated in Blast2GO. Overrepresented terms were considered if their enrichment fold change was >1.5 and the *p-*value was lower than 0.05. A bar chart was produced showing the *p*-values and fold changes for the top 20 most abundant GO terms among all enriched terms for each category of biological process, molecular function, and cellular components. We also use REVIGO^53^ to visualize the semantic relationships in highly redundant list of GO terms. The identified GO terms and their *p*-value were used as input in this tool. The GO terms have been clustered based on semantic similarity measure of SimRel and their frequenting calculated from whole UniProt database.

### RT-qPCR and MeRIP-RT-qPCR

Total RNA was extracted from Aag2 cells and treated with DNase I using TURBO DNase (ThermoFisher Scientific). cDNA (2μg) was synthetised using SuperScript™ III Reverse Transcriptase (ThermoFisher Scientific) followed by qPCR using QuantiFast SYBR Green PCR mix (Qiagen) in accordance with the manufacturer’s instructions. RT-qPCRs were carried out using a Rotor-Gene thermal cycler (QIAGEN). For qPCR, cDNA was diluted in 1:5 with UltraPure DNase/RNase-free water (Invitrogen). Two microlitres of the diluted cDNA was used in 10 μL qPCR reactions with both forward and reverse gene-specific primers (10 μM) with an initial 95 °C 5 min, and 40 cycles of 95 °C 20 s, 65 °C 15 s, 72 °C 10 s. Each reaction was run with at least three biological replicates, each with three technical replicates. The *RPS17* gene was used for normalizing data. Primer sequences are provided in Supplementary Table 2.

For MeRIP-RT-qPCR, EpiMark N^6^-Methyladenosine Enrichment Kit was first used to conduct MeRIP on total RNA harvested from DENV-infected Aag2 cells according to the manufacturer’s instructions (New England BioLabs). This was followed by RT-qPCR as above. Positive and negative controls for m^6^A were provided in the kit.

### RNAi-mediated gene silencing

For RNAi experiments, dsRNAs were synthesized in vitro using the T7 MEGAscript transcription kit according to the manufacturer’s instructions (ThermoFisher Scientific, USA). T7 promoter sequences were incorporated in both forward and reverse primers (Supplementary Table 2). For dsRNA synthesis, 200–500 ng of PCR product was used for each reaction. The reaction was incubated at 37 °C overnight, DNase-treated, and precipitated by the lithium chloride method following the manufacturer’s instructions. *F*our micrograms of the dsRNA was used to transfect Aag2 cells with 5 μl of Cellfectin transfection reagent (Invitrogen, USA). The cells were transfected again with the same reagent at 72 h after the first transfection. Cells were collected for RNA extraction as required for further analysis at 48 h after the second transfection.

### Treatment of cells with 3-DAA

The methylation inhibitor 3-Deazaadenosine (3-DAA; D8296, Sigma-Aldrich) was used to inhibit RNA methylation^54^. Aag2 cells were treated with 100 μM 3-DAA or as indicated in the text. Cells were collected for mRNA analysis at 48 h post-treatment.

### Measurement of RNA stability

Aag2 cells were plated in 12-well plates and then infected with DENV-2 at MOI 1. At 3 days post-infection, medium was changed to the 1:1 Mitsuhashi-Maramorosch and Schneider’s insect medium which contained 1 μM Actinomycin D (Sigma-Aldrich). After 4, 8, 12 and 16 h post-treatment, total RNA was extracted from the Aag2 cells using QIAzol lysis reagent (QIAGEN) and treated with TURBO DNase I (Thermo Fisher). The percent of *Ubc9* mRNA remaining was analysed through RT-qPCR.

### Overexpression of Ubc9 protein

The complete open reading frame for *Ubc9* was cloned downstream of the *GFP* gene in the plasmid vector pSLfa-PUb (Addgene) under the polyubiquitin promoter. For overexpression, 2 μg of the plasmid was transfected into Aag2 cells using the Cellfectin reagent (Invitrogen) according to the manufacturer’s instructions.

### Statistics and reproducibility

For all the statistical analyses and production of the graphs GraphPad Prism version 9 was used. Data were tested for normality using Shapiro–Wilk test. *T*-test was used to determine the significance levels between two samples, and One-way ANOVA was used to determine significance levels between three or more treatments. More details, including the number of replicates, are provided in the relevant figure legends. Data points on graphs represent *n* = biological replicates.

### Reporting summary

Further information on research design is available in the Nature Research Reporting Summary linked to this article.

## Supplementary information

Supplementary information

Supplementary Data 1

Supplementary Data 2

Supplementary Data 3

Supplementary Data 4

Reporting Summary

Description of Additional Supplementary Files

## Data Availability

MeRIP-Seq data have been deposited in the SRA under the accession PRJNA668808.
