# Supplementary material for: N6-methyladenosine modification of the Aedes aegypti transcriptome and its alteration upon dengue virus infection in Aag2 cell line
Source: Commun Biol. 2022 Jun 20;5:607. doi: 10.1038/s42003-022-03566-8 (PMC9209429; doi:10.1038/s42003-022-03566-8)
Supplement: Supplementary file 1 — Supplementary information [file 42003_2022_3566_MOESM1_ESM.pdf]

N^6-methyladenosine modification of the *Aedes aegypti* transcriptome and its alteration upon dengue virus infection in Aag2 cell line

Zhenkai Dai, Kayvan Etebari and Sassan Asgari*

Supplementary information

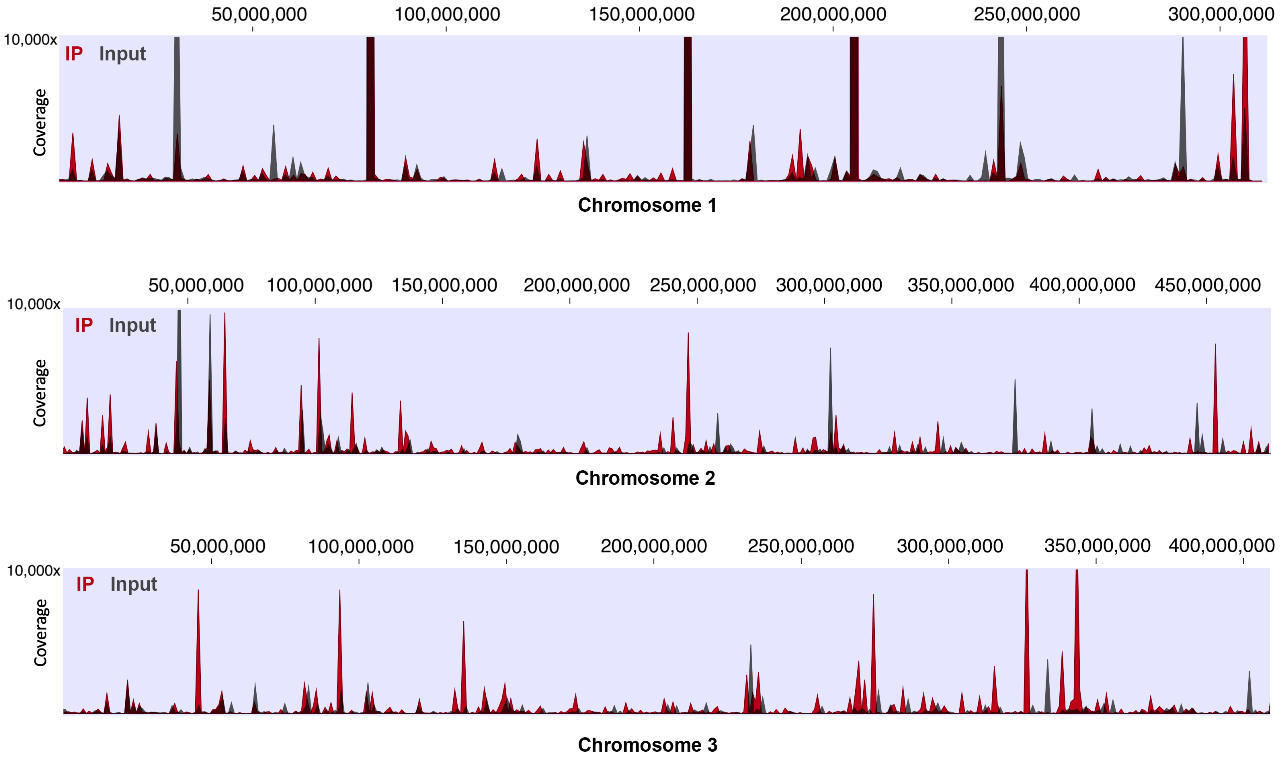

Supplementary Fig. 1: The reads distribution patterns from Input and IP groups at the *Aedes aegypti* chromosomal level. The read density chart determined by mapping MeRIP-Seq data of RNA isolated from Aag2 cells against AaegL5.0 genome reference.

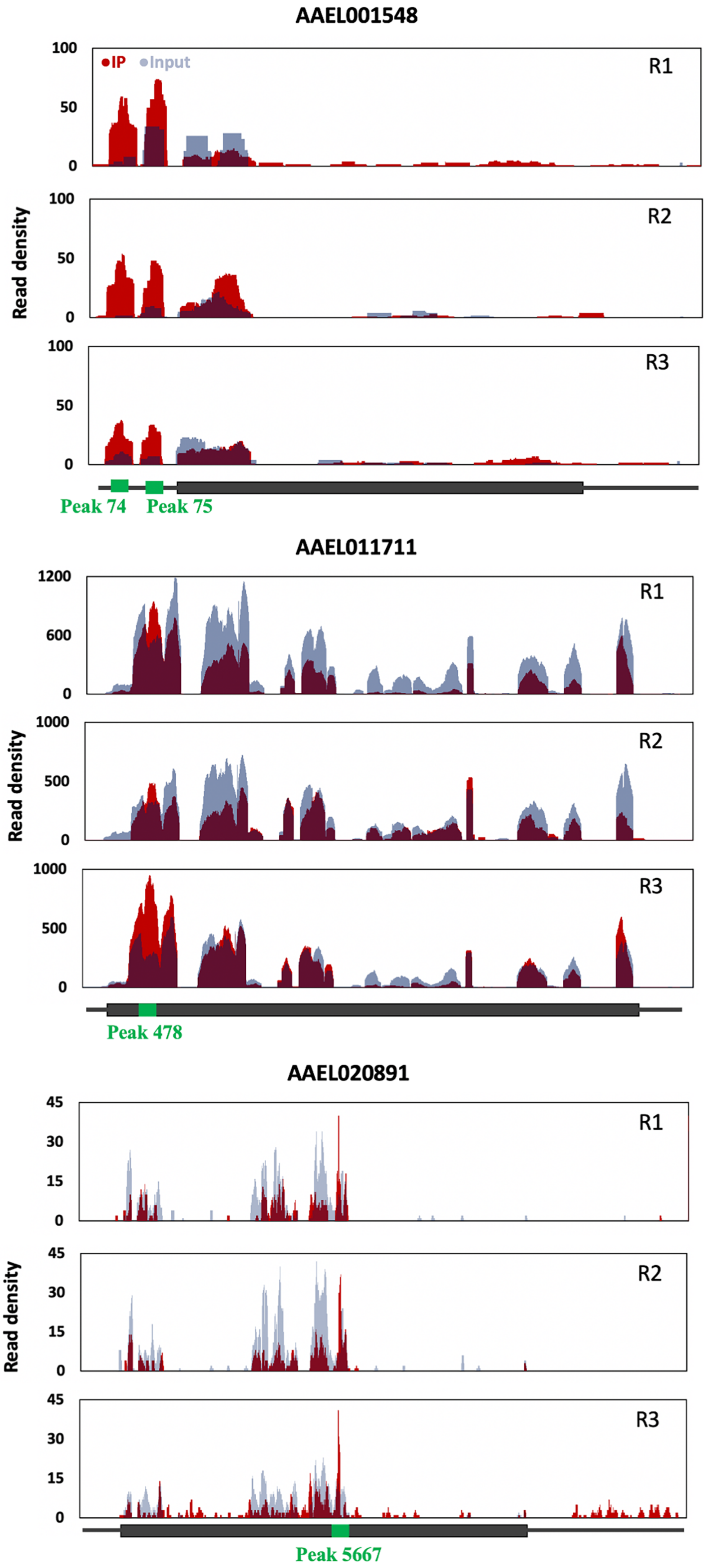

Supplementary Fig. 2: Distribution of m^{6}A peaks in Input and IP groups in three replicates for three representative *Aedes aegypti* genes with identified m^{6}A peaks determined by MeRIP-Seq of RNA isolated from Aag2 cells.

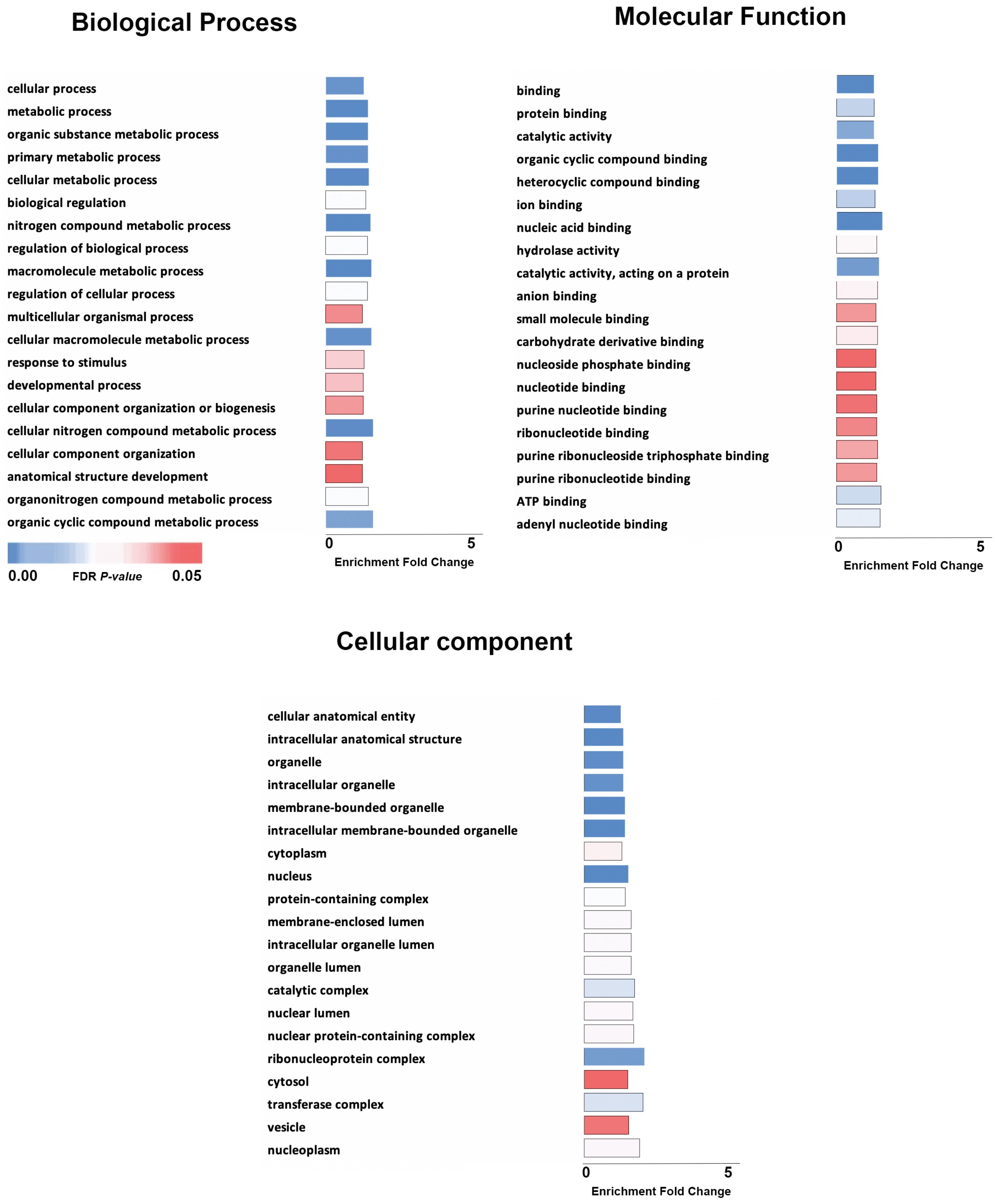

Supplementary Fig. 3: Gene ontology term enrichment analysis of *Ae. aegypti* transcripts with m^{6}A modification. Genes were classified into three categories of biological process, molecular function, and cellular component.

a

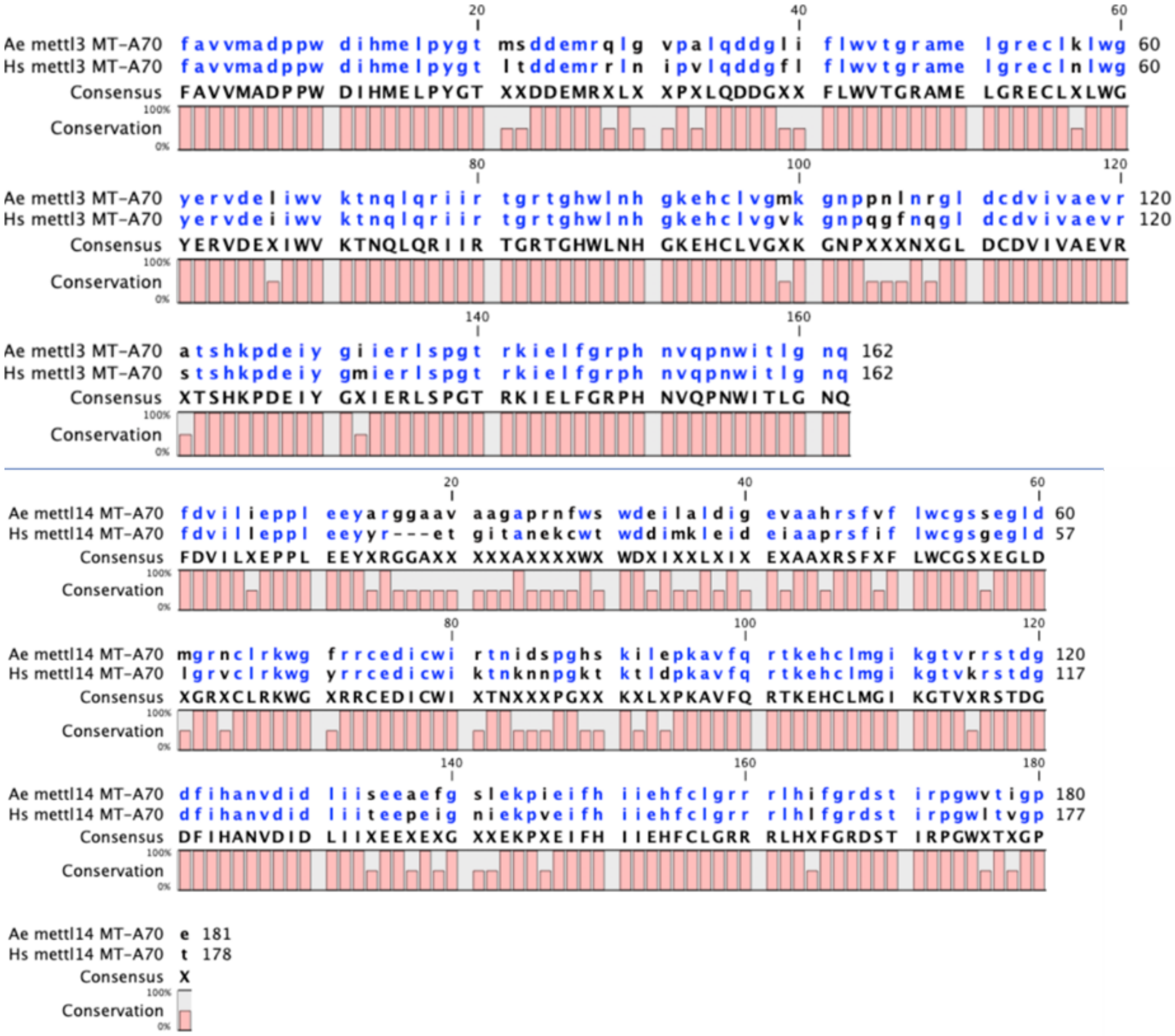

Ae mettl3 MT-A70
Hs mettl3 MT-A70
Consensus
Conservation
Ae mettl14 MT-A70
Hs mettl14 MT-A70

b

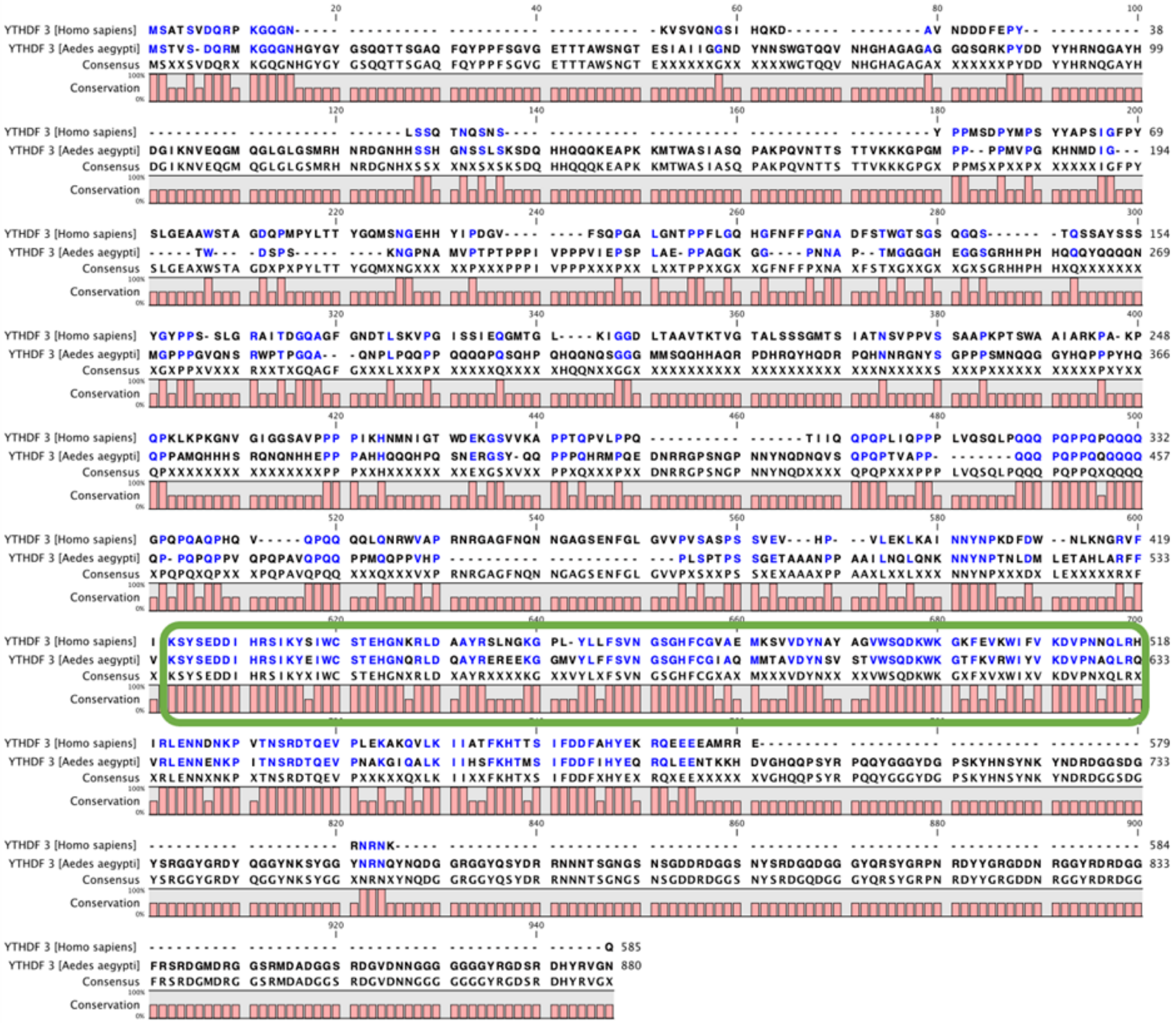

Supplementary Fig. 4. Sequence alignment of three m^{6}A related protein. a Amino acid sequence alignment of the MTA-70 domain of METTL3 and METTL14 proteins from *Homo sapiens* (Hs) and *Ae. aegypti* (Ae). **b** Amino acid sequence alignment of the entire YTHDF3 protein from *H. sapiens* and *Ae. aegypti*. The YTH domain is indicated by a green rectangle. All the alignments were generated using Clustal W algorithm of CLC Genomics Workbench software.

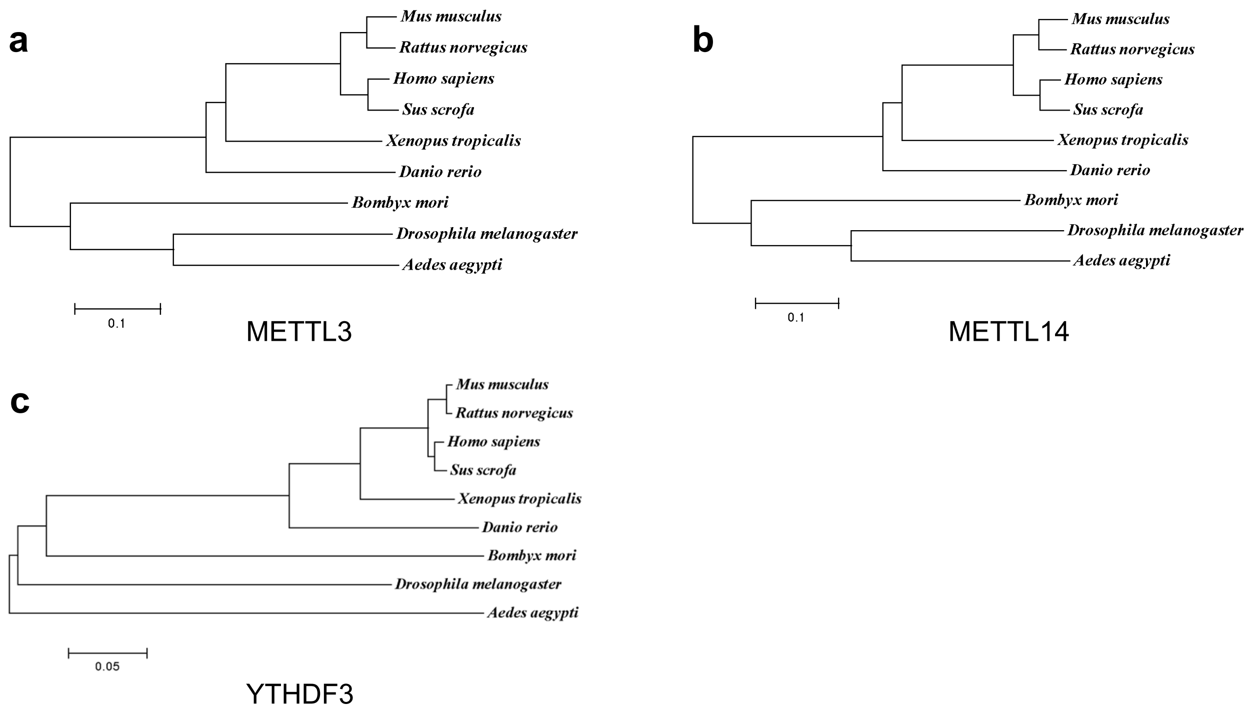

Supplementary Fig. 5. Phylogenetic trees of m^{6}A-modification-related genes. **(a)** METTL3, **(b)** METTL14, and **(c)** YTHDF3.

Supplementary Table 1. DENV-2 m^{6}A peaks and their positions in the virus genome.

	Start	End	Length	Coding region
Peak 1	540	789	249	pM
Peak 2	3460	3766	306	NS1-NS2A
Peak 3	7121	7868	747	NS4B-NS5
Peak 4	8778	8933	155	NS5
Peak 5	8957	9117	160	NS5

Supplementary Table 2. Primers used in this study.

qPCR Primers	Gene names	Primer sequences
AAEL026586F	uncharacterized protein LOC110676643 isoform X1	GGTAGAGGACGAGCGAATGG
AAEL026586R		ACCGGTTACTTCCGCATTGT
AAEL011112F	sorbitol dehydrogenase-like	CGGTGACTTCGTGGTTCAGA
AAEL011112R		CAAAAGTCGCAAACTCGGCA
AAEL020023F	histone H2B-like	GGAAAGGCCGCGAAGAAATC
AAEL020023R		CCAGTGTCGGGATGGACTTG
AAEL025779F	SUMO-conjugating enzyme UBC9-B	CCACTGTTCCACCCGAATGT
AAEL025779R		CAGCAGATCCTGAATGCCGA
MEETL3F	METTL3	ACATGGAGCTTCCTTACGGA
MEETL3R		GGCCAGTAACCCAAAGGAAT
METTL14F	METTL14	CTACGGACGGAGATTTCATC
METTL14R		ACGTCTTCCCAAGCAGAAAT
YTHDF-qF	YTHDF	GCAACAAGTGAACCACGGTC
YTHDF-qR		CCCTGCATTCCTTGCTCAAC
DENV-qF	DENV-2 NS1	GGTATGGTGGGCGCTACTA
DENV-qR		CAAGGCTAACGCATCAGTCA
RPS17-qF	Ribosomal protein subunit 17	CACTCCGAGGTCCGTGGTAT
RPS17-qR		GGACACTTCGGGCACGTAGT

RNAi primers	Primer sequences
AAEL026586RNAiF	TAATACGACTCACTATAGGGAGTAAGAAGCCGACGAGTGCTG
AAEL026586RNAiR	TAATACGACTCACTATAGGGAGCAGCATGCGGTCCATTTCAG
AAEL011112RNAiF	TAATACGACTCACTATAGGGAGCGGTCCGATTGGATTGGTGA
AAEL011112RNAiR	TAATACGACTCACTATAGGGAGCGTCGATTTTTCCACTCGCC
AAEL020023RNAiF	TAATACGACTCACTATAGGGAGCTGGCGTTTCTTCAAAGGCT
AAEL020023RNAiR	TAATACGACTCACTATAGGGAGTTCGCGGGAAGTGATTGTCG
AAEL025779RNAiF	TAATACGACTCACTATAGGGAGGCGTGCTAATCGGGGTAGTT
AAEL025779RNAiR	TAATACGACTCACTATAGGGAGAGGCGAGTTGGTTGTGAGAA
MEETL3_RNAi-F	TAATACGACTCACTATAGGGAGAAAAGCAAGATTCTCCTCACGGC
MEETL3_RNAi-R	TAATACGACTCACTATAGGGAGTTGGCTTCGTCAGCAGTTCCAGA
METTL14_RNAi-F	TAATACGACTCACTATAGGGAGCTACGTGAAAAGTCGCAGAAGCG
METTL14_RNAi-R	TAATACGACTCACTATAGGGAGGAGCGGCCACTTCACCAATATC

Sequences in red represent the T7 promoter sequences.
