# Supplementary material for: N6-methyladenosine modification of the Aedes aegypti transcriptome and its alteration upon dengue virus infection in Aag2 cell line
Source: Commun Biol. 2022 Jun 20;5:607. doi: 10.1038/s42003-022-03566-8 (PMC9209429; doi:10.1038/s42003-022-03566-8)
Supplement: Supplementary file 7 — Description of Additional Supplementary Files [file 42003_2022_3566_MOESM7_ESM.pdf]

Description of Additional Supplementary Files

File name: Supplementary Data 1
Description: m6A peaks identified in Aedes aegypti through MeRIP-Seq analysis

File name: Supplementary Data 2
Description: Gene Ontology enrichment of Aedes aegypti genes with m6A peaks

File name: Supplementary Data 3
Description: Differentially expressed m6A peaks due to dengue virus infection

File name: Supplementary Data 4
Description: Differentially expressed genes due to dengue virus infection
